# Genome-Wide Discriminatory Information Patterns of Cytosine DNA Methylation

**DOI:** 10.3390/ijms17060938

**Published:** 2016-06-17

**Authors:** Robersy Sanchez, Sally A. Mackenzie

**Affiliations:** Department of Agronomy and Horticulture, University of Nebraska, Lincoln, NE 68588, USA

**Keywords:** epigenetics, epigenomics, information thermodynamics, linear discriminant analysis, machine learning

## Abstract

Cytosine DNA methylation (CDM) is a highly abundant, heritable but reversible chemical modification to the genome. Herein, a machine learning approach was applied to analyze the accumulation of epigenetic marks in methylomes of 152 ecotypes and 85 silencing mutants of *Arabidopsis thaliana*. In an information-thermodynamics framework, two measurements were used: (1) the amount of information gained/lost with the CDM changes $I_{R}$ and (2) the uncertainty of not observing a SNP $LC_{R}$. We hypothesize that epigenetic marks are chromosomal footprints accounting for different ontogenetic and phylogenetic histories of individual populations. A machine learning approach is proposed to verify this hypothesis. Results support the hypothesis by the existence of discriminatory information (DI) patterns of CDM able to discriminate between individuals and between individual subpopulations. The statistical analyses revealed a strong association between the topologies of the structured population of *Arabidopsis* ecotypes based on $I_{R}$ and on *LC_R_*, respectively. A statistical-physical relationship between $I_{R}$ and $LC_{R}$ was also found. Results to date imply that the genome-wide distribution of CDM changes is not only part of the biological signal created by the methylation regulatory machinery, but ensures the stability of the DNA molecule, preserving the integrity of the genetic message under continuous stress from thermal fluctuations in the cell environment.

## 1. Introduction

Cytosine DNA methylation (CDM) is one of the molecular processes that result in epigenetic modifications to the genome. Specifically, cytosine methylation is a widespread regulatory factor in living organisms, and changes introduced by DNA methylation can be inherited from one generation to the next. Some methylation changes can regulate gene expression and effect genomic imprinting [1,2]. Cytosine methylation arises from the addition of a methyl group to a cytosine’s C5 carbon residue. Distinct pathways regulate methylation status by the action of methyltransferases [3]. The addition or removal of a methyl group to a cytosine C5 residue produces a change of information that is recognized by the molecular transcription machinery and can be verified by current sequencing technologies [2]. However, it is still unclear whether or not the observed methylation changes are linked to genome-wide information patterns.

The development of DNA bisulfite conversion methodology coupled with next-generation sequencing approaches (Bis-seq) allows determination of the methylation status of nearly every cytosine in a genome. In this way, the methylation status of particular cytosine sites is often expressed in terms of methylation level *p_i_* = *#C_i_*/(*#C_i_* + *#nonC_i_*,), where *#C_i_* and *#nonC_i_* represent the numbers of methylated and non-methylated read counts observed at the genomic coordinate i, respectively. At a tissue level, the methylation status (methylated or non-methylated) of cytosine $C_{i}$ at the genomic coordinate i can be analyzed as a random variable that takes value “methylated” with probability $p_{i}$ and “non-methylated” with probability $1-p_{i}$. However, at a tissue level, the measurement of the methylation status at every single cytosine site carries an amount of uncertainty.

Uncertainty in a given system is exposed when more than one unknown event may occur. Thus, uncertainty as “a state of incomplete information” [4] can be expressed in terms of probabilities, *i.e.*, as a real number between 0 and 1 [5]. In our case, at a tissue level, the uncertainty of methylation status at each individual cytosine site can be quantitatively expressed by means of the methylation level or by means of the entropy of the methylation level [6,7]. In particular, we are interested in the genome-wide pattern of uncertainty variations or, more specifically, the amount of information gained after an uncertainty reduction in the methylation status at each single cytosine site. Thus, information about the methylation status is expressed as difference in entropies, before and after a methylation change [8].

The physics of information (expressed as difference in entropies) is expressed by Landauer’s principle, according to which a molecular machine must dissipate a minimum energy of $\varepsilon =\;k_{B}T\;\ln\;2$ (about 3 × 10^−21^ Joules at room temperature) at each step in the (genetic) logic operations including proofreading [8,9]. Theoretically, Landauer’s principle is a consequence of the second principle of thermodynamics [8]. The experimental demonstration of information-to-energy conversion was published in 2010 [10], while Landauer’s principle was experimentally verified in 2012 [9]. The biophysical foundation of the information involved in DNA methylation processes expressed as difference in entropies before and after a methylation change (denoted here as *I_R_*) was recently shown [11]. In this last work, the authors proposed a statistical mechanical model that allowed the estimation (consistent with experimental data) of a basic mechanical property of the DNA molecule: the *DNA persistence length*. Their results were also consistent with the measured role of the DNA persistence length in methylation processes. Evidence suggests that methylated ds-DNA has a substantially higher persistence length than non-methylated DNA [12], and its effect increases the rigidity of the DNA molecule as well as nucleosome compaction and rigidity [12,13].

The physics of information and the molecular biophysics of CDM processes raise the question of whether or not uncertainty variation in genome-wide CDM changes induced by environmental variation creates footprints of information patterns in individual methylomes. Here, we show results indicating that genome-wide information patterns are revealed by the uncertainty variation of methylation status at specific methylation regions (landmarks) on chromosomes. Multivariate statistics and machine learning approaches are applied to detect discriminatory information (DI) genomic regions able to distinguish between individuals and between individual subpopulations. We suggest that the DI patterns of CDM not only reflect the ontogenetic history of each individual, but are responsive to stability of the DNA molecule. The current work is not intended to represent all the possible analyses, but proposes a new methodology based on information theory concepts, which are founded on the statistical mechanics of CDM (see reference [11]), and on the application of machine learning approaches. Our approach does not exclude others currently in use, but enriches the analytical arsenal for understanding methylation modes.

## 2. Results

Our study was accomplished in a novel information-thermodynamics framework for methylome analysis where two magnitudes were used: (1) the amount of information gained/lost ($I_{R}$) with the CDM changes processed by the methylation machinery in a genomic region (GR) *R* (Equation (1), see material and method section) and (2) the uncertainty of not observing a SNP ($LC_{R}$, Equation (3)). The physical basis of the amount of information $I_{R}$ has been described in the Introduction and is documented in a recent publication [11]. The physical foundation of $LC_{R}$ is given in Section 4.4. That is, Equations (1) and (3) permit the quantification of physical count data in terms of information-thermodynamic magnitudes. Results presented here center on the hypothesis that epigenetic marks are chromosomal footprints accounting for different ontogenetic and phylogenetic histories of individual populations. These histories are embodied in the topology of the population structure, which is analyzed based on the application of a machine learning approach and statistical analyses.

The research involves analysis of methylome and SNP datasets reported for 152 *Arabidopsis* ecotypes in a published study by Schmitz *et al.* [14], with methylome data from 86 silencing mutants taken from a study published by Stroud *et al.* [15]. In all the datasets, individual samples are given by summarized count data (details about these datasets are in Materials and Methods section). The existence and accumulation of epigenetic marks was analyzed in methylomes of the 152 ecotypes and 86 silencing mutants based on estimation of $I_{R}$ (Equation (1)), while the existence of SNP marks was analyzed based on the estimation of $LC_{R}$ (Equation (3)).

For downstream analysis, we applied a machine learning approach to derive the topology of the structured population of *Arabidopsis* ecotypes. Results indicated striking similarities between the topologies based on $I_{R}$ and on $LC_{R}$. An analogous approach was applied to the 86 silencing mutants to test whether or not methylation patterns reflect biological links between mutated genes, detectable by the machine learning approach applied.

Selection of genomic features (GFs) based on different machine learning approaches and used for classification purposes led to the identification of gene sets that appear to be involved in environmental adaptation. Based on our results, feature selection and feature extraction were required steps in searching for DI methylation patterns able to discern between individuals and individual subpopulations. The discriminatory power of the selected genomic features is then evaluated based on the performance of a reference machine learning classifier. This goal is not fully attainable within the current state of the art, involving analysis based on *ad hoc* concepts of differentially methylated positions (DMPs) and differentially methylated regions (DMRs).

### 2.1. The Hotspots of Methylation and SNP Landmarks

**Figure 1:**
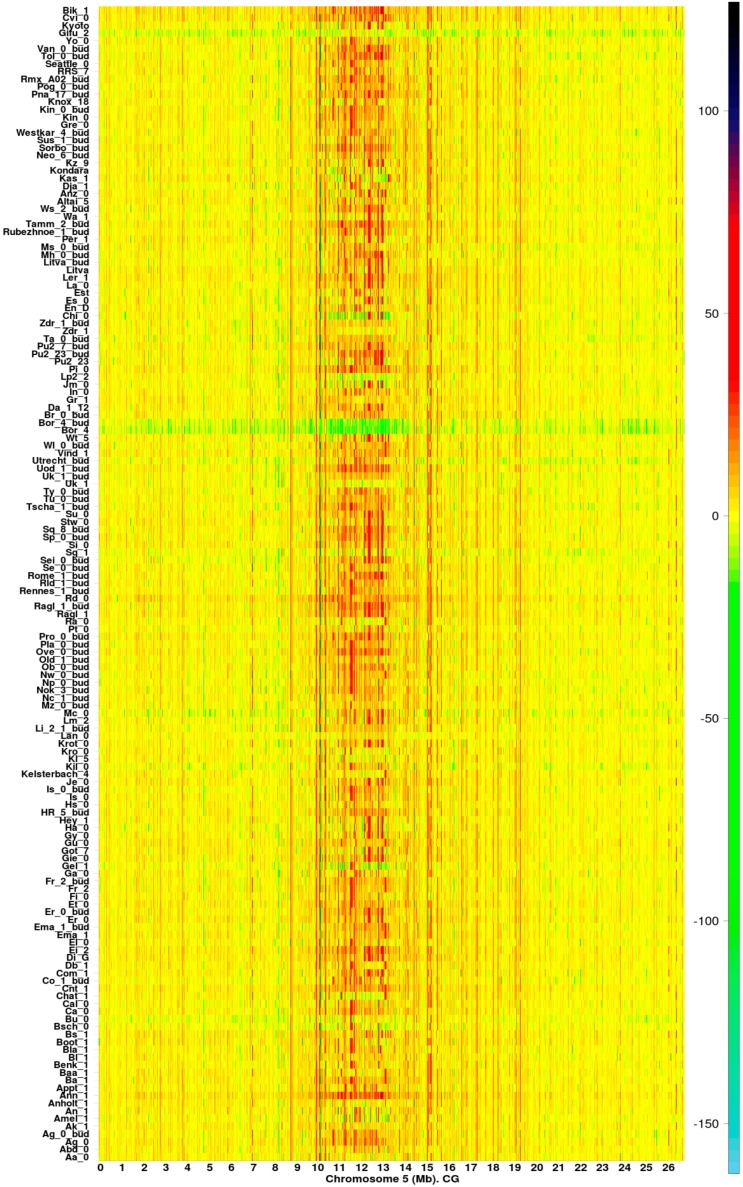
Methylation hotspots along chromosome 5 from 151 *Arabidopsis* *thaliana* ecotypes [14] (CG methylation context). The color bar indicates the magnitude of $I_{R}$ values.

**Figure 2:**
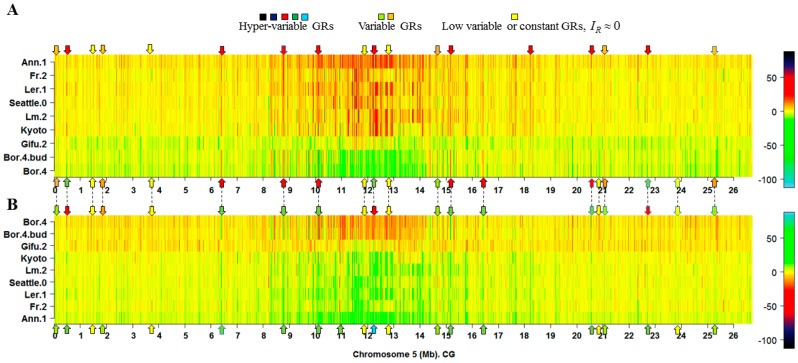
Illustrative heatmap showing the classification of GRs into hypervariable (HMRs), variable (VMRs) and low-variable or constant (LMRs) methylated regions. (**A**) The maximum and minimum of the $I_{R}$ values correspond to black and sky blue, respectively; (**B**) The same samples, but with inverted color scale, equivalent to the photograph negative; the maximum and minimum correspond to sky blue and black, respectively. The heatmap for all the ecotype samples is given in Figure S16. In general, HMRs are regions with $\left|I_{R}\right|>>0$ . In both panels, A and B, the HMRs readily visible are those straight lines in orange to black colors. In panel A, HMRs are GRs with $\left|I_{R}\right|>>0$ and in panel B are those GRs with $I_{R}<<0$ . In both panels, the arrows in red, green, and sky blue indicate that at least one HMR is found in the observed heatmap position. The arrows in orange and light green indicate that at least one VMR is found in the specified heatmap position, while arrows in yellow indicate that at least one LMR is present. It must be noticed that LMRs are the most abundant types of GRs. The apparent abundance of HMRs results from the compression of sample vectors for 13,370 GRs. As a result, some GRs are superimposed in the graphic. In the present example, only 12,971 from 13,370 × 9 = 120,330 GRs (11% ) have $\left|I_{R}\right|\geq 10$ bit. A quantitative way to define the borders of each class can be set by applying fuzzy set and fuzzy logic theory, beyond the limit of the current work.

Estimations of $I_{R}$ revealed the existence of methylation hotspots along chromosomes (Figure 1 and Figures S1–S4, CG contexts). Genomic regions (GRs) can be classified across the samples according to the value of $I_{R}$ as: (1) highly variable methylation regions (HMRs); (2) variable methylation regions (VMRs); and (3) low variable or constant methylation regions (LMRs). This classification is illustrated in the heatmap presented in Figure 2. The classification of the GRs into HMRs, VMRs, and LMRs must not be confused with the classification of individual methylomes, which is presented below in Section 2.1 and Section 2.2.

Regions with information gain (orange to black on the heatmap color bar) or loss (light yellow to sky-blue) (Figure 1 and Figures S1–S4) are observed at specific positions, with a high density in the pericentromeric region. Lines in yellow correspond to regions where the difference in entropies $H_{R}^{ecotype}$ and $H_{R}^{Col-0}$ is close to zero. GRs with $I_{R}>0$ were, in general, more abundant than GRs $I_{R}<0$. According to Equation (1), methylation hotspots are ecotype chromosomal regions with remarkably high uncertainty variation with respect to Col-0. In particular, methylation hotspots experience significant decreases in the absolute value of information $I_{R}$ ($\left|I_{R}\right|$).

**Figure 3:**
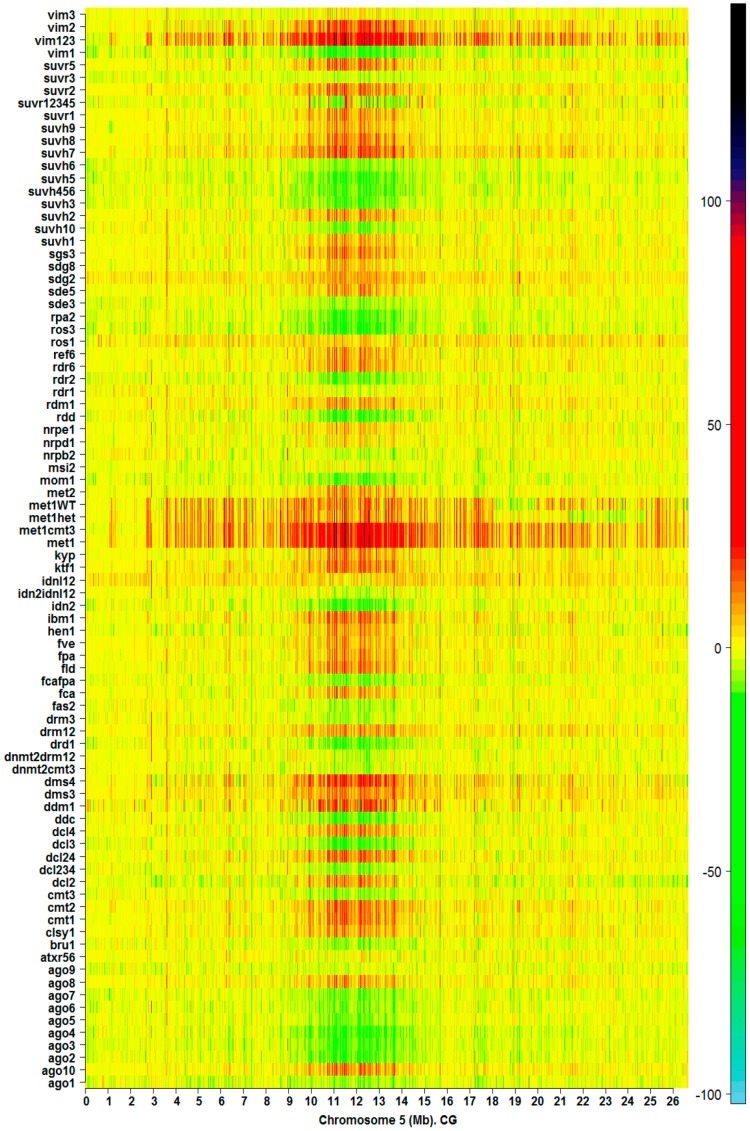
Methylation hotspots along chromosome 5 from 83 *Arabidopsis* silencing mutants in CG context. The color bar indicates the magnitude of $I_{R}$ values (Equation (1), Material and Methods).

Landmarks of $I_{R}$ methylation hotspots are also observed in heatmaps of silencing mutants in both methylation contexts CG and CHG. Mutation of genes associated with methylation processes range in magnitude of effect on the natural landmarks observed in the ecotypes. The heatmap of $I_{R}$ for CG methylation on Chromosome 5 from 85 silencing mutants is presented in Figure 3. Similar heatmaps for the remainder of the chromosomes (CG and CHG methylation contexts) are shown in Figures S5–S9. Depending on the mutant, dysfunction in the methylation machinery can create a distinctive pattern of landmarks on the chromosome. However, with the exception of primary methylation determinants like *met1*, *ddm1*, and *vim123*, a significant number of $I_{R}$ methylation hotspots are preserved relative to the corresponding wild type in both methylation contexts CG and CHG. In addition, Figure 3 and Figures S5–S13 show that, with perhaps a few exceptions, mutation of a gene directly involved in the methylation pathways for one context, CHG or CG, does not affect the other.

**Figure 4:**
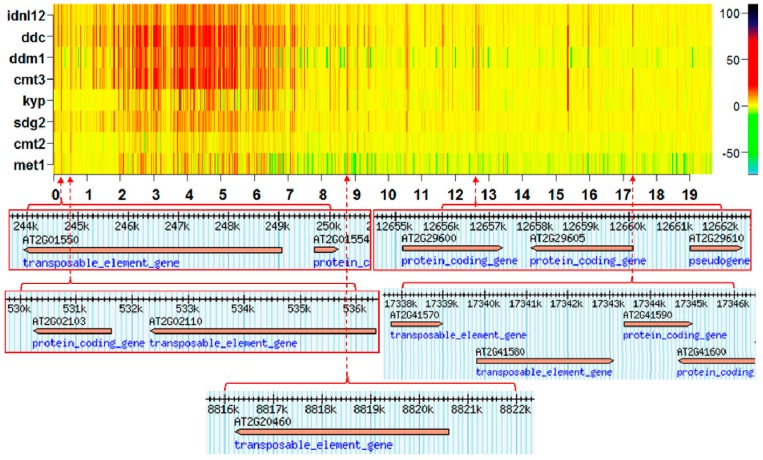
Annotation of several CHG methylation hotspots on chromosome 2 from eight *Arabidopsis* silencing mutants.

The annotation of methylation hotspots suggests that landmarks consistently affected by methylation changes frequently target transposable elements (TEs), TE genes, and pseudo-genes. An example of this is presented in Figure 4. These results are consistent with earlier experimental observations that TEs are primary targets of the methylation machinery [15,16].

**Figure 5:**
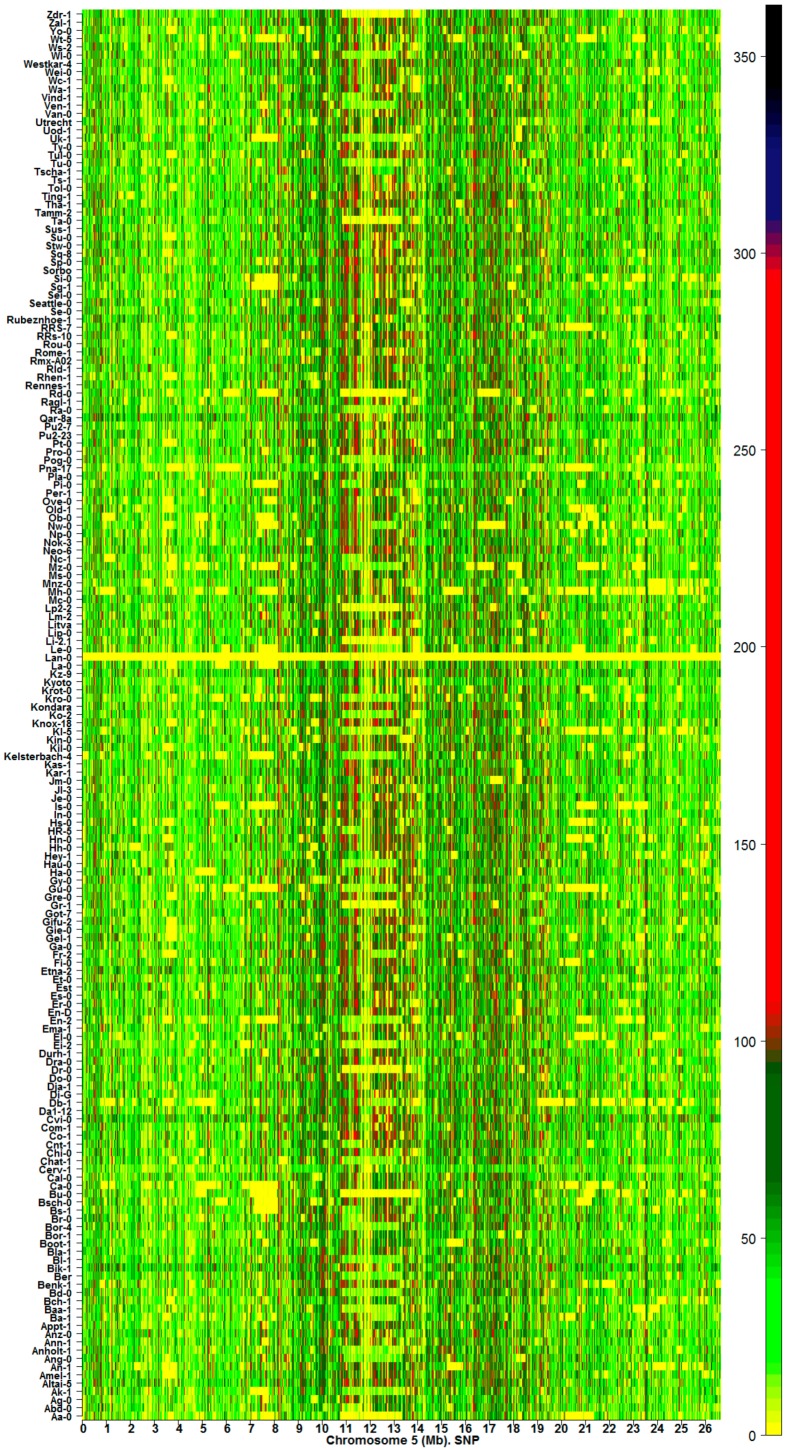
Mutational hotspots along chromosome 5 from 83 *Arabidopsis* silencing mutants. The color bar indicates the magnitude of $LC_{R}$ values (Equation (3), Material and Methods).

Landmarks of mutation hotspots along the chromosomes are revealed by *LC_R_* heatmaps (Figure 5). The landmarks are clearly distinguished as highly variable (red/black) regions along chromosomes and across samples. The heatmaps indicate that GRs with *LC_R_* can be classified according to the level of base substitution into (1) highly variable regions; (2) variable regions; and (3) low variable or constant regions. Therefore, it is possible to distinguish between individuals and among subsets of individuals by considering their DI and mutational patterns.

### 2.2. Discriminatory Information Patterns in Natural Arabidopsis Ecotypes

**Figure 6:**
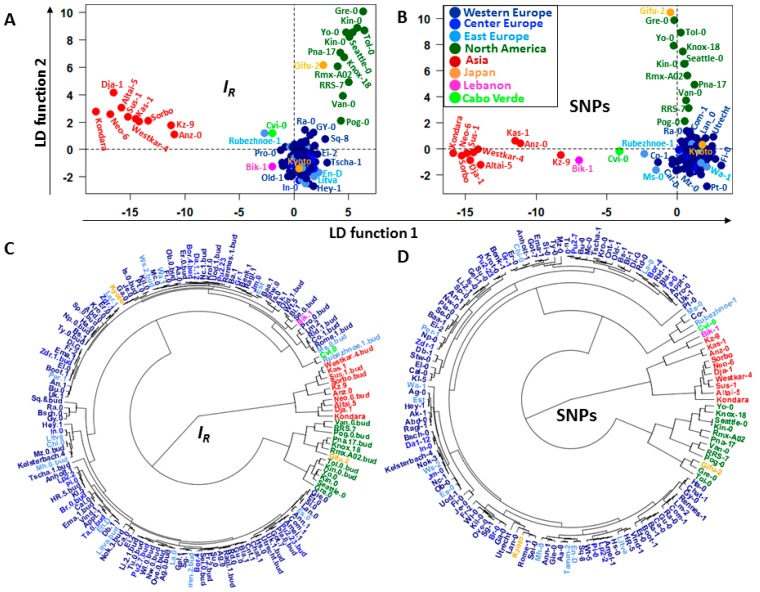
Classification of the *Arabidopsis* ecotypes according to their geographical distribution. (**A**,**B**) LDAs based on $I_{R}$ and $LC_{R}$ (SNPs), respectively; (**C**,**D**) fan dendrograms based on the individual coordinates estimated from the linear discriminant (LD) functions. The dendrograms were built by applying hierarchical clustering with Euclidean distance and UPGMA as agglomeration method.

Although the epigenomic diversity the heatmaps suggest the existence of specific landmark informative patterns in all chromosomes across the ecotype samples that may or may not be shared by several individuals. These patterns comprise chromosomal regions carrying DI. After applying hierarchical clustering based on levels of C-DMRs, Schmitz *et al.* [14] showed that of the 151 *Arabidopsis thaliana* ecotypes analyzed, those from North America and Asia reflected their geographical distributions. However, consecutive application of principal component analysis (PCA) and linear discriminant analysis (LDA) of the same ecotype set in this study supports the hypothesis that identified landmark patterns can better account for the ontogenetic and phylogenetic differences among individuals (Figure 6A,C).

The analysis supports ecotype classification by geographical location not only for North America and Asia [14], but for virtually all geographical regions except for the Japanese ecotypes Gifu-2 and Kyoto, which are grouped together with North America and European ecotypes, respectively. Ecotype classifications were conducted by methylome footprints, and also by single nucleotide polymorphism (SNP) patterns detected across ecotype genome sequences (Figure 5 and Figure 6B,D). *Arabidopsis thaliana* ecotype classification consistency with their geographical distribution was striking between landmark methylation patterns and SNP patterning (Figure 6). A summary of the classification results is presented in Table 1.

The analysis was focused on CG methylation context. It appears that the three methylation contexts of CG, CHG, and CHH (where H=A, T or C) may have distinct biological roles in *Arabidopsis* [3]. Primary genomic sites for differential CHG and CHH methylation are not gene regions, but more often transposable element and repetitive sequences. Thus, analysis in CG methylation permitted us to assess the effect on classification of a significant proportion of gene region methylation.

**Figure 7:**
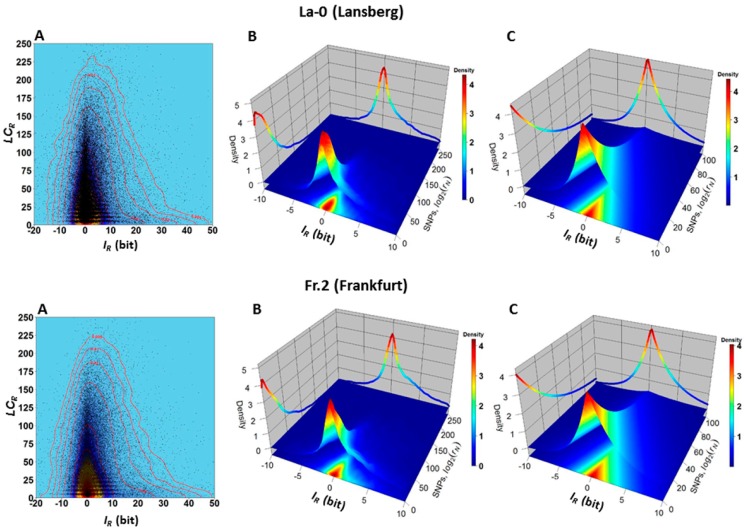
Dependence between variables $I_{R}$ and $LC_{R}$ in the ecotypes La-0 and Fr.2. (**A**) The 2D kernel density plots $I_{R}$ *versus* $LC_{R}$ indicate that most $LC_{R}$ values are located in a narrow band around the vertical line $I_{R}=0$ . That is, the density plots expose a statistical tendency: most of the GRs with lower uncertainty variations (lower methylation changes) also experience, in accordance with Equation (5), a lower uncertainty level (SNP not observed), determined by a lower probability that an SNP is present within a GR; (**B**) The 3D kernel density plot indicates that, for example, with high joint probability $P\;\;\left(-1\leq I_{R}\leq 1,\;\;0\leq LC_{R}\leq 25\right)$ (the volume of the prism with squared base formed by the intervals $-2\leq I_{R}\leq 2$ and $0\leq LC_{R}\leq 25$ and truncated by the surface, which covers red to yellow region) genomic regions *R* with values $-2\leq I_{R}\leq 2$ and $0\leq LC_{R}\leq 25$ are observed. For these regions there is a low probability of observing SNPs (in accordance with Equations (4) and (5) and a low value of normalized counts supporting SNPs in the regions Equation (3). In another example, with low joint probability $P\;\;\left(-1\leq I_{R}\leq 1,\;\;150\leq LC_{R}\leq 200\;\right)$ (corresponding to the volume of the prism truncated by the surface with squared base in the intervals $-1\leq I_{R}\leq 1$ and $150\leq LC_{R}\leq 200$), genomic regions *R* with values $-1\leq I_{R}\leq 1$ and $150\leq LC_{R}\leq 200$ are observed; (**C**) 3D plot of the density probability distribution of the Farlie–Gumbel–Morgenstern copula built from the non-linear fit of the marginal distributions estimated for $LC_{R}$ (a Weibull PDF) and $I_{R}$ (a Skew–Laplace PDF). The existence of a structural dependence between the variables, $I_{R}$ and $LC_{R}$ is suggested by the Farlie–Gumbel–Morgenstern copula distribution [17,18], which describes in an acceptable approach the empirical behavior shown in panel B. That is, the stochastic relationship between the uncertainty variation of methylation levels (Equation (1)) and the uncertainty of not observing a SNP (Equations (3) and (5)) in a GR is confirmed. These estimations were performed for several *Arabidopsis* ecotypes. The results for the ecotypes La-0 and Fr.2 are shown.

The similarity between hierarchical clusters suggests that some statistical-physical relationship may exist between the SNPs and methylation changes. The two-dimensional (2D) and three-dimensional (3D) kernel density plots presented in Figure 7 support this hypothesis. The 2D kernel density plots indicate that the frequency of normalized read-counts supporting SNPs decreases with the increment of methylation changes, expressed here as gain or loss of information $I_{R}$ (Figure 7A). The empirical 3D kernel density plots (Figure 7B) indicate the existence of a non-trivial relationship between the uncertainty variations of methylations levels and the uncertainty level to observe a SNP in a GR. This last statement implies the existence of a structural dependence between the variables $I_{R}$ and $LC_{R}$. This dependence is supported by the Farlie–Gumbel–Morgenstern copula distribution built from non-linear fit of the marginal distributions (Figure 7C).

### 2.3. Discriminatory Information Patterns in Silencing Mutants

**Figure 8:**
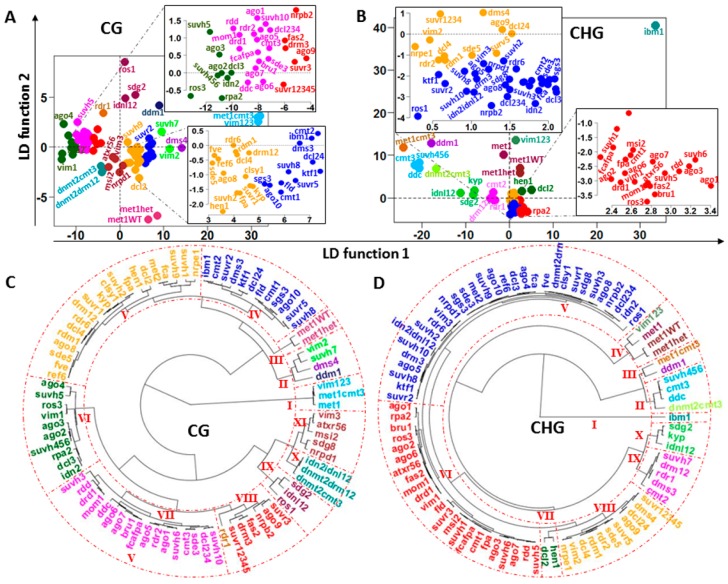
Classification of silencing mutants based on DI regions. (**A**,**B**) LDAs based on $I_{R}$ estimated for CG and CHG methylation contexts, respectively; (**C**,**D**) fan dendrograms based on the individual coordinates estimated from the LD functions. The dendrograms were built by applying hierarchical clustering with Euclidean distance and UPGMA as agglomeration method. Roman numbers identify the main clades.

Depending on the mutant, dysfunction in methylation machinery leaves a distinctive pattern of landmarks on the chromosomes. The mutants can be divided into subsets based on their peculiar footprints for $I_{R}$. A prior classification was derived by the consecutive application of PCA (to reduce dimensions) and hierarchical cluster to the whole set of mutants. The consecutive application of PCA, LDA and hierarchical cluster (using the mutant coordinates in the LD functions) to the $I_{R}$ vectors permitted grouping into subsets (Figure 8). Existence of genome-wide methylation patterns of DI was validated by applying LDA and SVM in several variants (see Table 2).

Next, for both methylation contexts, CG and CHG, 9428 DI regions were extracted based on their correlation with the first three and first two PC components, respectively. As presented in Table 2, the validation results support the premise that these regions carry sufficient DI to divide the set of mutants into different subsets according to the posterior classification presented in Figure 8.

### 2.4. GF Selection and the Topology of the Dendrograms of Structured Populations

**Figure 9:**
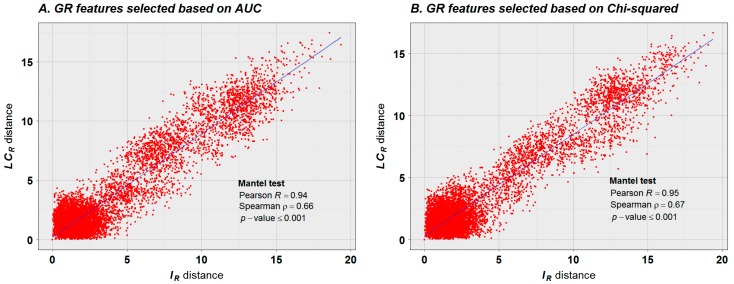
Relationship between the distance matrices estimated for variables *I_R_* and $LC_{R}$. The distance matrices were estimated for the ecotypes represented as vectors of the selected GFs. (**A**) the selection of GFs was based on the classification performance of each GR expressed in terms of AUC. In this case the features selected for $I_{R}$ do not overlap with those selected for $LC_{R}$; (**B**) the selection of GFs was based on the classification performance of each GR expressed in terms of Chi-squared statistic. In this case the matrices were built with the intersection of GR features selected for $I_{R}$ and $LC_{R}$. However, similitudes between topologies derived for the population structure based on $I_{R}$ and $LC_{R}$ remain consistently high independent of the GF set selected, as reflected in the graphic and Mantel test results. This explains the semblance between the dendrograms presented in Figure 6.

The step of GF selection in our analysis revealed that different subsets of relevant genomic features with discriminatory power to properly discern the topology of the structured population can be retrieved based on different machine learning attribute selection algorithms. This result is presented in Figure 9. The strong linearity between the ecotype distances based on $I_{R}$ and $LC_{R}$ are visually marked and highly statistically significant.

To analyze the spectrum of biological processes simultaneously affected by methylation and SNP linkage to the topology of the *Arabidopsis* ecotype population, a GO enrichment analysis of genes within the selected GFs was accomplished. We analyzed 477 annotated protein-coding genes within GF, selected based on the Chi-squared value. As shown in Table 3 and Figure S14, several significant GO terms were detected (full details in Table S1). Since enrichment analysis consists of multiple steps with multiple assumptions for its application, we applied a Fisher exact test to gene sets from GO terms not reported significant (0.05 < *p*-values < 0.1) in the entire analysis (performed with *runTest* function from the R package *topGO* [19]). That is, we tested the enrichment of genes from specific GO terms with respect to the whole set of protein-coding genes found in GR features. Results revealed gene enrichments from three GO terms that can play a fundamental role in environmental adaptation: (1) GO:0034641: cellular nitrogen compound metabolic process; (2) GO:0009733: response to auxin stimulus; and (3) GO:0006950: response to abiotic stress, response to biotic stress. The list of genes from this analysis is in Tables S2–S4. A list with genes from GO:0006950 (response to abiotic stress, response to biotic stress) is given in Table 4.

A rough estimation of the discriminatory power of *LC_R_* with respect to *I_R_* (and *vice versa*) in GR features derives from the rate of Chi-squared statistics $\chi_{LC_{R}}^{2}/\chi_{I_{R}}^{2}$. A greater value of the Chi-square statistic indicates greater discriminatory power to discern between individual subpopulations.

The histogram in Figure S14 suggests, however, that although the rate is statistically greater than 1, on average the rate is not far from 1. So, although the discriminatory power from methylation is also present in GRs with $\chi_{LC_{R}}^{2}/\chi_{I_{R}}^{2}>1$, it can be expected that, for the subset of genes inside these GRs, the balance of discriminatory power is slightly tilted toward the effect of SNPs in the region. Results of gene enrichment analysis for the set of genes under the restrictions $\chi_{LC_{R}}^{2}/\chi_{I_{R}}^{2}>1$ and $\chi_{LC_{R}}^{2}/\chi_{I_{R}}^{2}<1$ are presented in the Tables S2 and S3. The gene enrichment analysis for genes inside of selected GFs from the GO:0034641 (cellular nitrogen compound metabolic process) and genes from the GO:0009733 (response to auxin) are given Tables S5 and S6.

## 3. Discussion

Methylation hotspots shared by a set of individuals at fixed chromosomal positions suggest the existence of specific landmarks of DI (Figure 1, Figure 2 and Figure 3). That is, most of the CDM changes observed in natural variation and silencing mutants occur at specific methylation GRs, which are delineated in the heatmaps as chromosomal landmarks. The effect of the silencing mutants on methylation is revealed in the heatmaps of *I_R_* as distinctive footprints, where a considerable number of the landmarks observed in ecotype samples are intensely modified in the mutants. The greatest intensity of methylation changes occur in pericentromeric and centromeric regions, which are rich in TEs, TE genes, and pseudo-genes (Figure 1, Figure 2 and Figure 3 and Figures S5–S13). In general, CHG landmarks consistently found on chromosome arms frequently covered TE-related sequences with some in protein-coding regions (Figure 4). This observation suggests that CHG landmarks may be associated with two main functions linked to methylation changes: the prevention of TE activity and expression, and gene silencing [20].

Depending on the silencing mutant, different scales of disrupting effect on two methylation hotspots, CG and CHG, were observed. Since variations in *I_R_* values quantitatively express gain or loss of information along the chromosome, patterns of methylation hotspots observed in the heatmaps reflect the magnitude of *de novo* reprogramming induced by the silencing mutations. In particular, the heatmaps suggest that methylation processes traditionally linked to CHG context are not independent of those linked to CG context. These observations appear to be consistent with recent findings by other groups that reflect an overlap in methylation regulation between these two contexts [15,20,21].

It is worth noting that according to Equation (1), the range of observable values of *I_R_* depends on the size of the regions, which derives from the fact that *I_R_* is a linear function of the entropy difference at each single cytosine position included in the GR. In consequence, different information patterns can be revealed within different region sizes. That is, the analysis of the information patterns of CDM carried out in our study is not limited to specific GR size, and the machine learning approach proposed here can be applied at different fixed GR sizes. For the purpose of discovering DI patterns with the application of machine learning methods, the partition into genomic regions requires not fixed GR sizes, but consistency across the samples. The GRs could be limited to genomic sections of biological interest. A particular application of our approach could be performed, for example, to partition the methylome into potential word frameworks (PWFs), as proposed in reference [11]. PWFs are binary stretches (clusters) of methylation marks. It was shown that about 75% of the normalized counts of PWFs in *Arabidopsis* comprise methylation signals concentrated in gene regions [11]. The study of discriminatory information patterns in methylome partitions into PWFs is a relevant subject for further studies.

### 3.1. Simlarities of Discriminatory Information Patterns in Silencing Mutants May Reflect Biological Relationships between Them

A further step in the detection of DI patterns requires the application of clustering and machine learning algorithms. Although the classification performed by machine learning algorithms mainly reflects similarities or differences between final genome-wide methylation profiles induced by the mutants, some grouping of silencing mutants would also indicate their relationship within the regulatory methylation network. For several genes involved in methylation processes, the observed groupings appear to be consistent with their roles in methylation pathways. In clade-II for CHG methylation context (Figure 8D), linkage of suvh456, cmt3, and ddc is consistent with literature reporting CMT3 as a primary CHG methyltransferase in *Arabidopsis* [3], while histone methyltransferases KYP/SUVH4, SUVH5, and SUVH6 are shown to be required for CMT3-dependent CHG methylation [20,22] (symbols for wildtype genes are given in uppercase letters and their mutational variants in lowercase). Hypomethylated DMRs from *kyp suvh5/6* and *ddc* (the triple mutant *drm1drm2 cmt3*) overlap in 89.5%, and the triple mutant emulates the effect of *cmt3* [15]. Hence, the grouping of mutants *cmt3*, *ddc*, and *suvh456* into a subset is expected. This subset is part of a larger conglomerate of mutants encompassing clades II and III, which include *dnmt2cmt3*, *ddm1*, and *met1cmt3*, each of which is a separate cluster. However, distances between mutants from clades II and III are smaller than the distance between any one of them and the remainder of the mutants. This observation is consistent with known biological relationships between the members of clade-II. Mutation in *DDM1* disrupts CHG methylation, and loss of DNA methylation occurs in sites regulated by *KYP/SUVH4*, *SUVH5*, and *SUVH6* [15].

Inherited methylation DI patterns were detected by the LDA analysis. In plants, CG methylation is maintained by *methyltransferase 1 (MET1*). The inherited CG methylation background of *met1*^+/−^ heterozygous progeny *met1*^+/+^ (met1WT) and met1^+/−^ (methet) located them to clade III (Figure 8C). Landmarks in the CG heatmaps indicate that *met1*^+/−^ progeny do not recover the CG methylation status of the original wild types (Figure 3 and Figures S5–S9), since mutations of *MET1* result in elimination of CG methylation throughout the genome. This is in agreement with the Stroud *et al.* (2013) conclusion that genic methylation is severely impaired; the progeny plants of *met1*^+/−^ displayed morphological defects that led them to investigate their methylome [15]. However, the inherited CHG methylation DI patterns of *met1*, methet, and met1WT located them in clade IV of the CHG dendrogram (Figure 8D).

Another interesting relationship is suggested by the members of clade I in the CG cluster (Figure 8C). The mutants *met1*, *met1cmt3*, and *vim123* are able to introduce an extensive *de novo* reprogramming of DNA methylation along chromosomes in both methylation contexts CG and CHG (Figure 3 and Figures S5 to S13). These mutants are members of clades III (*met1cmt3*) and IV (*met1* and *vim123*) in CHG context. *MET1*, controlling maintenance of CG methylation, also requires three *variant in methylation* family proteins: VIM1, VIM2, and VIM3. In the *vim1vim2vim3* triple mutant, a global loss of DNA methylation in CG context that strongly resembles the methylation profile in *met1* mutants is observed [15] (Figure 3 and Figures S5–S8). VIM1, VIM2, and VIM3 have overlapping functions in maintenance of global CG methylation and epigenetic transcriptional silencing [23].

Clade-X from the CHG dendrogram insinuates a less obvious relationship (Figure 8D). The double mutant *idnl1/2* closely emulates the disruption produced by mutants *sdg2* and *kyp*, two set domain proteins involved in epigenetic control of gene expression with histone methyltransferase H3-K4 and H3-K9 specificity, respectively (Figure 8D and Figures S9–S13). *IDN2* together with either *IDNL1* or *IDNL2* is required for complete *DRM2*-mediated genome methylation [24]. In *Arabidopsis*, *de novo* methylation of any cytosines in CG, CHG, and CHH (H = A, T, or C) is initiated by *domains rearranged methyltransferase 1* (*DRM1*) and *DRM2*. During siRNA induced transcriptional gene silencing, *IDN2* together with *IDNL1* or *IDNL2* are recruited and DNA methylation accomplished by DRM2, followed by removal of active chromatin marks and by H3K9 methylation [25]. Mutational effects of *sdg2* and *idnl1/2* are positioned relatively closely in the CG cluster analysis, integrating clade IX (Figure 8C). In addition, the observation from the dendrograms and heatmaps in Figure 1, Figure 3 and Figures S5–S13 indicate that the effects of *idn2* and the triple mutant *idn2/idnl1/2* are less severe than for *idnl1/2*. In particular, differences between the effects of *idn2* and *idn2/idnl1/2* mutants in CHG methylation (clade V) are smaller than in the CG context (clades VI and X, respectively). This observation suggests that alternative processes in the methylation pathways may mitigate to some extent the disrupting effect caused by the absence of *idn2* complex with *idnl1* or *idnl2*.

At least three RNA-dependent RNA polymerases (RDR1, RDR2, and RDR6) are functional in plants in the siRNA silencing pathways [26]. It has been reported that DCL4 is the primary processor of endogenous RDR6-dependent trans-acting siRNAs (tasiRNAs) [27]. However, DCL2, DCL3, and perhaps DCL1 were able to produce RDR6-dependent short interfering RNA (siRNAs) in the absence of DCL4 [27,28]. Clade-V from the CG dendrogram (Figure 8C) suggests, however, that the disrupting effect produced by mutants *rdr6* and *dcl4* (clade V) on CG methylation are not so different, indicating that the contribution of DCL1, DCL2, and DCL3 may not be sufficient to fully bypass the absence of DCL4.

The effects of mutant *dcl2* (clade V) and the double mutant *dcl2/4* (clade IV) are closer to *dcl4* than *dcl3* (clade-VI), while the *hen1* mutant effect is quite close to *dcl2*, supporting a relationship already reported. HEN1 has been shown to participate in DCL2-mediated antiviral defense, influencing survival of virus-infected plants at high temperatures [29]. The main contribution to the triple mutant *dcl234* (clade-VII) seems to be from *dcl3*. DCL3 functions with RDR2 to form chromatin-associated siRNAs (24 nucleotides) required for DNA methylation guided through AGO4 [30]. The siRNA–AGO4 complex may bind complementary DNA and thereby define the region to be methylated by DRM2, previously recruited by AGO4 [31,32]. However, DCL2 and DCL4 functionally compensate for the effect of mutant *dcl3* [27]. This report appears to be consistent with the large distance between the effects of mutants *rdr2* (clade VII, CG, and clade VIII, CHG) and *dcl3* (clade VI, CG and clade V, CHG) on both CG and CHG dendrograms (Figure 8C,D). Nevertheless, *dcl3* and *ago4* mutants are grouped in the same clade in both methylation contexts, and their effects on CHG methylation context are very close.

The purpose of the above discussion is not to illustrate the “rediscovering” of already known relationships, but to show that the mutation of genes involved in the methylation process leaves footprints of methylation patterns that reflect biological links (if any) between the corresponding genes. These footprints and the biological links reflected by them are detectable by machine learning methods, which is an indication of the capacity of this approach to discover new knowledge.

### 3.2. Links between the Discriminatory Informational and Mutational Patterns in Natural Arabidopsis Ecotypes

Multivariate statistical analysis suggests a relationship between landmarks of methylation and mutation hotspots. In particular, classifications of the *Arabidopsis* ecotypes based on *I_R_* and *LC_R_* do not show significant difference in delineating geographical regions of Asia, Europe, and North America. This observation implies that divergence arising during the natural mutation process in a structured population might be influenced by regulatory methylation mechanisms. This statement finds support in recent reports of cytosine methylation effects on DNA mechanical properties, affecting DNA flexibility and stability [12,33,34,35,36]. Figure 6 suggests a strong relationship between population structure based on methylation and SNPs at GR level, inferred from the variables *I_R_* and *LC_R_*, respectively. It is notable that the Japanese ecotypes Gifu-2 and Kyoto were misclassified together with subsets of North American and European ecotypes in our study and in the hierarchical clustering reported for CG-SMPs and SNPs by Schmitz *et al.* (Supplementary information from reference [14]). The consistent grouping of the Japanese ecotypes with the North American and European regions in both analyses implies early adaptive steps of convergent molecular evolution [37]. At a molecular level, convergent evolution is linked to similar SNP patterns that lead to identical replacements of single amino acids within the encoded product of a protein-coding gene occurring independently in unrelated taxa [38,39]. The plausibility that this type of evolutionary tendency could be observed in isolated individuals from the same lineage is presumably higher than between individuals from different lineages.

The classification results presented here reflect significant progress in deriving methylome relationships relative to previous analyses [14]. The progress is shown in three key aspects: (1) classification results are consistent with the geographical regions for 149 of 151 available methylomes of *Arabidopsis* ecotypes (Figure 6), and is not limited to subsets from Asian and North American groups. (Figure 2e,f in reference [14]). Previous analysis methods produced misclassification for the European ecotypes Ei-2 and Vind-1, and several European ecotypes were misclassified together with Asian and North American ecotypes; (2) In the method presented here, classification rested on supervised classifiers, and all classifiers were validated (Table 1), with classification accuracy persistently high; (3) The correlation between the distance matrices reported earlier (Table S5 from [14]) suggests a weak relationship between the population structure based on methylation data and SNPs. The best result reported by the authors yielded a Spearman correlation coefficient of 0.4. In the present study, we used Mantel’s test to compare the distance matrices. The result, based on 7000 permutations, reported Pearson and Spearman correlation coefficients above 0.94 and 0.66, respectively (Figure 9), suggesting a strong linear relationship between the topologies of the population structure based on methylation data and SNPs at GR levels.

### 3.3. Consistent Topologies of the Population Structure Based on $I_{R}$ and $LC_{R}$

As suggested by the results presented in Figure 9, more than one set of GFs with discriminatory power to distinguish between the individual populations can be found (based on AUC, Figure 9A, and based on Chi-squared, Figure 9B), and topologies of the population structure based on $I_{R}$ and $LC_{R}$ remain strongly consistent. The fact that more than one set of GFs with discriminatory power can be detected is not surprising based on molecular marker studies; frequently more than one set of molecular markers (used to represent individuals as vectors) can be used to estimate a consistent topology of populations. In our case, however, not only molecular-genetic markers are under consideration, but also epigenetic markers and, as presented in Figure 9, their effects on the topology of the population structure is pronouncedly in concert.

The low correlation between methylation and SNPs reported in reference [14] could be expected if the regulatory CDM changes are signals from an epigenetic communication system, as proposed in reference [11]. In this scenario, genome-wide CDM methylation changes would not show strong correlation with SNPs unless filtering the signal from the methylation background noise. However, the reported correlation of 0.4 in reference [11] is not too small and it is significant. The reason for that resides in that this correlation was not derived from arbitrary genome-wide methylation data, but from DMPs in the CG methylation context; *i.e.*, the original data was previously filtered. In an analogous way, the results presented in Figure 6 and Figure 9 do not derive from arbitrary genome-wide methylation data, but the methylation signal has been filtered by applying a an elaborated machine learning approach, which increased the signal-to-noise ratio by removing the GRs with low discriminatory power.

The classification results presented in Figure 6, Figure 8 and Figure 9 are based on a previous step of feature selection, which detects the GRs with greater discriminatory power. This step is necessary, since adaptation to a new environment does not imply entire methylome reprogramming, but specific genomic regions may be regulated. Usually, many GRs under regulatory control are correlated (originating redundant information), and their contribution to the epigenomic response could be quantitative (epiQTLs). A recommended way to deal with this situation is to perform a further step of feature extraction by applying, for example, PCA. The discriminatory power of this approach is reflected in Figure 6, Figure 8 and Figure 9 (see Table 1 and Table 2). The approach is not limited to the application of LDA and SVM, but many other classifiers can be applied, such as those available in data mining software Weka [40].

Downstream analysis, which follows the machine learning steps, permits the identification of potential biomarkers that could play a role in the adaptation of *Arabidopsis* ecotypes (Figure S14, Table 3 and Table 4 and Tables S1–S6). For example, the list of genes within selected GFs from GO:0006950 (response to abiotic stress, response to biotic stress) presented in Table 4 suggests that the combination of feature selection, feature extraction, and machine learning classifier steps leads to meaningful *in silico* identification biomarkers. *Arabidopsis* ecotypes included in the current analysis cover geographical regions from the Mediterranean to the north of Europe. The enrichment of genes with GO:0006950 is consistent with the range in light intensity, precipitation, and heat confronted by these ecotypes across their environments.

One could argue that a feature selection approach should cover GRs greater than that covered by the primary transcripts. Since mutations and methylation can alter the local 3D DNA shape, the stability of nucleosomes and, thus, affect the local chromatin structure, their effect goes beyond the simple nucleotide base position [2]. For example, mutations or methylation changes that alter the 3D shape of a transcription factor DNA-binding site in the proximal promoter region of a gene will alter expression of a gene that is sometimes located a few hundred bases downstream. However, transcription factor binding sites can also occur at greater distances upstream, in introns or even downstream of target genes. DNA mutations in the cognate binding site can reduce or abrogate the affinity between transcription factor and DNA [41]. Cytosine methylation in the binding site can increase local rigidity of the DNA molecule [12,13] and prevent bending of the DNA around the transcription factor [41].

### 3.4. Potential Role of Methylations on the Fixation of New Mutations

Recent reports support that most of the CDM changes observed in natural conditions serve to stabilize the DNA molecule and conform to statistical mechanical principles [11]. Thus, CDM can play a role in the local stabilization of new random mutational events [42,43,44]. This hypothesis is supported by Figure 7. The 2D and 3D kernel density plots suggest that most of the observed CDM changes tend to preserve the integrity of the message carried by the DNA molecule, which is challenged by thermal fluctuations in the cell environment. CDM changes can alter the mechanical properties of the DNA molecule to maintain its stability [33]. Thus, a statistical-physical relationship between CDM changes and SNPs is expected. Indeed, depending on DNA sequence context, the addition or removal of a methyl group to a cytosine residue could increase or decrease the local thermodynamic stability of DNA and its nucleosomes [33,36,45,46,47].

Assuming a non-role of methylation in stabilizing the DNA molecule, randomness of thermal fluctuations and occasional environmental changes would yield no dependence at all between variables $I_{R}$ and $LC_{R}$. The structural dependence between the variables $I_{R}$ and $LC_{R}$ is revealed by the Farlie–Gumbel–Morgenstern (FGM) copula distribution [17]. Although FGM copula quantitatively expresses a weak dependence [18], one could argue that this is expected, since in normal natural conditions most of the GRs are compacted and protected in the nucleosomes [48] and methylation is not required in this state to stabilize the DNA molecule [48,49].

The FGM copula distribution presented in Figure 7 suggests that the SNPs occurring at GR and are not statistically independent from CG methylation. Panels B and C indicate that with high probability (the volume under the surface) GRs with $I_{R}$ values around zero ($\left|I_{R}\right|\approx 0$) carry the smaller values of $LC_{R}$. Therefore, in accordance with Equation (5) and Figure 7, the greater uncertainty values for SNP occurrence are found with high probability in GRs with $\left|I_{R}\right|\approx 0$. In other words, a value $\left|I_{R}\right|\approx 0$ is an indicator of GRs less likely to experience SNP events.

Experimental evidence indicates that CDM plays an important role in preserving the stability of DNA [12,43,50,51,52]. As a consequence, adaptation of an individual to a new environment can be expected to induce regulatory methylation responses (biological signals) that would likewise ensure DNA stability. These would be frequent methylation changes that could vary from cell to cell in the same tissue. CDM changes that are induced by random thermal fluctuations form the simplest natural explanation to observed “spontaneously occurring variation” for DNA methylation in *Arabidopsis* plants propagated by single-seed descent through multiple generations [53,54].

In an evolutionary context, the combined results presented in Figure 6, Figure 7 and Figure 9 suggest that high values of uncertainty variation $\left|I_{R}\right|$ (e.g., in the interval $4\leq I_{R}\leq 10$ in Figure 7) could be created by the methylation machinery to stabilize the DNA molecule affected by new mutations ($50\leq LC_{R}\leq 200$) and to reduce the probability of further mutational events. This is reflected in Figure 9, observing that most of the distances between individuals are less than 4 bits for both variables, $I_{R}$ and $LC_{R}$, while a strikingly linear tendency is revealed for distances greater than 4 bits. The apparent loss of association between the observed distances in the interval from 0 to 4 bits also has its explanation in an evolutionary context and in the dynamics, on a daily and seasonal basis, of CDM changes. An important subset of CDM changes regulates the process of gene expression and functional adaptation to the environment [47]. These are specific molecular signals from the regulatory methylation machinery. According to Figure 6, the patterns of mutation and methylation of individuals from closely related environments are very similar. In consequence, for these individuals most of the CDM changes will be transgenerational noise, mainly linked to local environmental variations and to the ontogenetic development of each individual. That is, the relationship between $I_{R}$ and $LC_{R}$ distances is revealed when subpopulations from different environments are taken into consideration.

**Figure 10:**
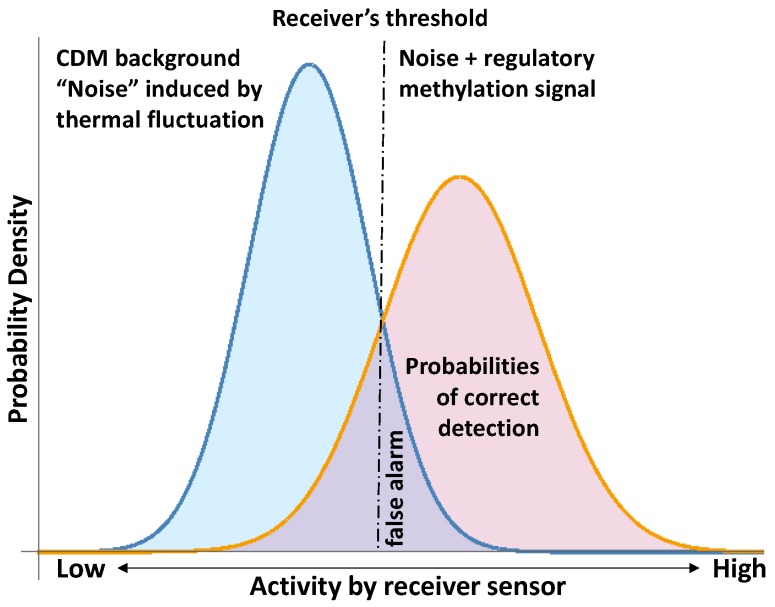
Signal detection in noise according to reference [57,58] and, here, applied to the detection of regulatory CDM signals.

The methylation changes addressed to preserve the stability of DNA molecules are stochastic methylation “background” noise with respect to the regulatory methylation signals. The challenge is to sort out the regulatory methylation signals from the CDM noise induced by thermal fluctuations and random mutational events. This signal-to-noise challenge has already been confronted (see [11,55,56,57]), and a concrete application in the context of CDM is illustrated in Figure 10. It is not possible to fully separate the regulatory methylation signal from the CDM background; even a simple regulatory methylation change could alter the mechanical properties of the DNA molecule [2,33,36] and thus could require additional local readjustment. Therefore, the receiver (a device to detect the signal) must set up a criterion for response; in this case, a threshold level of activity in its sensor (*i.e.*, a function of the methylation levels). This threshold, in combination with the PDF for noise and signal plus noise, determine the probabilities of correct detection [55,56] (Figure 10). Hence, any statistical analysis of the regulatory signals of CDM must consider the statistical thermodynamics subjacent to the methylation process.

## 4. Materials and Methods

### 4.1. Information Gain of a DNA Sequence Region R

Assuming that, as a result of variations in environmental conditions, a change of methylation status in a genomic region *R* takes place, the uncertainty decrease in the genomic region *R* leads to a gain (or loss) of information given by:

(1) $$I_{R}=-\left(\sum_{i\in R}H\left(C_{i}^{after}\right)-\sum_{i\in R}H\left(C_{i}^{before}\right)\;\right)$$

where $H\left(C_{i}^{after}\right)$ and $H\left(C_{i}^{before}\right)$ stand for Shannon’s entropy of the methylation status before and after the variations of environmental conditions, respectively [7,8], which is given by:

(2) $$H\left(C_{i}\right)=-p\left(C_{i}\right)\;log_{2}p\left(C_{i}\right)-\left(1-p\left(C_{i}\right)\right)\;log_{2}\;\left(1-p\left(C_{i}\right)\right)$$

and $p\left(C_{i}\right)=p_{i}$ is the methylation level at the genomic coordinate i, as mentioned in the introduction. That is, entropy defined by Equation (2) is the expected value of the logarithm base 2 of the methylation level [7]. Equation (1) expresses an information theoretical derived concept with a thermodynamic and biophysical meaning [8,59]. Equation 1 was used to compute the $I_{R}$ for several samples with methylation data available in online databases (see below).

### 4.2. Arabidopsis thaliana Methylation and SNP Data

According to Equation (1), $I_{R}$ is computed for a subject sample with respect to a given reference sample. The $I_{R}$ values were computed for 152 *Arabidopsis* ecotypes, which were generated by Schmitz *et al.* study [14]. The tabulated separated values (TSV) files taken from NCBI GEO under accession GSE43857 [14] were read and transferred to R software version 3.2.1 [60] by using the Bioconductor (version 2.14) R-package *GenomicFeatures* [61]. Ecotype Col-0 was used as a reference (152 ecotypes including Col-0). The read counts for each single cytosine position, reported in the TSV files, were used to compute $I_{R}$. Each TSV file reports for each cytosine: chromosome, position, strand, methylation context, numbers of methylated reads (*#C_i_*), and total numbers of reads (*#C_i_* + *#nonC_i_*,).

The *Arabidopsis* ecotypes SNPs data were downloaded from 1001 Genomes Data Center (http://1001genomes.org/datacenter/; or http://1001genomes.org/data/Salk/releases/; or http://signal.salk.edu/atg1001/download.php. TSV files). Each TSV reports for each mutations: chromosome, position, reference base, substitution base, quality, number of non-repetitive reads supporting substitution, and concordance. For all the samples we used only the reported SNPs with a quality score of 25 and above. Details about the pipelines for the generation of methylation and SNP data are given by Schmitz *et al.* study [14]. These authors used ecotype Col-0 as reference genome in both types of analyses, methylome and SNPs. So, to preserve consistency, we took the same reference, *i.e.*, the same sample Col-0.

The $I_{R}$ also was computed for 86 silencing mutants and the corresponding wild-type samples from a recent study from Stroud *et al.* [15] (GEO accession numbers GSE39901). The mutant ros1 and its corresponding wild type from [62] were also considered (GEO accession GSE33071). In the cases of these mutational studies, we used the methylation levels reported by the authors in the wiggle files.

### 4.3. Machine Learning Approach

To test the hypothesis that different environmental conditions must leave different landmark patterns on chromosomes, a machine learning approach was followed. Samples were represented as *N*-dimensional vector of *N* GRs (whole genome) with the corresponding $I_{R}$ estimated values.

The estimation of the area under the ROC curve (AUC) for the current multiple-class classification problem was performed according to reference [63] and applied to reduce the space dimension and to detect potential discriminant informative regions. This method was applied by using the R-package *HandTill2001* [63]. Principal component analysis (PCA) was also used to reduce space dimensions, from *N*-dimensions (whole genome) to a number of principal component carrying at least the 80% of the whole sample variance.

Independent feature selection was performed by using attribute evaluation based on Chi-squared statistic, which evaluates the worth of an attribute by computing the value of the Chi-squared statistic with respect to the class. This algorithm is implemented in Weka software [40] and applied here by using the R package *FSelector* [64]. The implementation of this algorithm includes Fayyad and Irani’s MDL method for supervised discretization [65].

AUC and PCA outputs were used with two classifiers: linear discriminant analysis (LDA) and support vector machine (SVM). These computations were performed by using the R-packages *adegenet* [66] and *e1071* [67], respectively. Although these machine learning approaches were applied genome-wide, they can be applied by chromosome or even by large chromosome sections as well, but the analysis must be applied consistently across the samples.

### 4.4. Logarithm of the Normalized Reads Counts

For a given number of non-repetitive reads supporting the base substitution *r*, the normalized reads counts $r_{N}$ were estimated as $r_{N}=r\;Concordance$, where *Concordance* stand for the read ratios supporting a predicted feature to the total coverage. The numerical values, *r and Concordance*, for each presumable SNP base position, are given in the mentioned TSV files from 1001 Genomes Data Center (see above).

Next, the sum of logarithm base 2 of DNA-base substitution counts at a given region R was computed as:

(3) $$LC_{R}=\sum_{i\in R}log_{2}\left(r_{N_{i}}\right)$$

In order to understand the physical foundation of the magnitude $LC_{R}$, we propose the analysis of fixed mutational events in a molecular biophysical context, where every mutational event has an energetic cost. It is naturally expected that the probability $p_{i}$ that an SNP is present at a single nucleotide position increases with $LC_{i}=log_{2}\left(r_{N_{i}}\right)$. As a matter of fact, the logarithmic transformation of read counts is used to stabilize variation between different datasets, which permits testing differences between the set of samples assuming normal distribution. That is, the physical differences between individual read counts are better revealed in the log-count-space, which implies a better distinction between the thermodynamic states of the system. So, from a thermodynamic point of view, the cost of fixing a mutation in the individual population must be proportional to $LC_{i}$. Then, under the assumption that the probability $p_{i}$ that a SNP is present at a single nucleotide position is given by the probability $P_{i}\left(LC\leq LC_{i}\right)$ to observe a value LC lesser than or equal to $LC_{i}$, the relationship between $p_{i}$ and $LC_{i}$ can be expressed by the equation:

(4) $$p_{i}=P_{i}\left(LC\leq LC_{i}|\beta ,\lambda \right)=1-\lambda \;e^{-\beta \;LC_{i}}\;\;(LC_{i}>0)$$

Hence, the probability $q_{i}$ that a single nucleotide position does not experienced a mutational event decreases with $LC_{i}$ according with the Boltzmann distribution $q_{i}=\lambda \;e^{-\beta \;LC_{i}}$ (5), where λ is the partition function of the thermodynamic system implicit in the biophysical context, β is a constant that is function of the temperature that include Boltzmann constant, and $p_{i}+q_{i}=1$. Next, from Equation 4, it follows that $LC_{i}$ expresses the uncertainty of not observing a SNP in a single nucleotide position according with the equation:

(5) $$\;LC_{i}=-\frac{1}{\beta }log\left(\frac{1-p_{i}}{\lambda }\right)$$

Since the probability $p_{i}$ is linked to a fixed mutation, it must not be confused with the probability to observe a mutational event at a single nucleotide position across the genome, which frequently follows a Poisson distribution. The non-linear regression analysis indicated that Equation (4) fit the ecotype experimental data used in our study. The cumulative distribution functions (CDF) given by Equation (4) and the P-P plots for the *Arabidopsis* ecotypes Seattle.0 and La.0 are presented in Figure S15.

### 4.5. Heatmaps

The numerical scale for each heatmap of *I_R_* is close to the range of values estimated from the experimental data according to Equation (1). The gradient of color scale is then constrained, in each case, to the maximum and minimum numerical values of *I_R_*. For example, in Figure 1, a sky blue color corresponds to the minimum value of *I_R_* on chromosome 5 from 151 *Arabidopsis* ecotypes, which is found significantly below the −100 bit, while black color corresponds to the maximum value of *I_R_*, which is found below the 100 bit. In Figure 3, sky blue and black are also assigned to the maximum and minimum values of *I_R_*, respectively. However, the range of *I_R_* variation in Figure 3 is wider than in Figure 1, which is consistent with the fact that extreme methylation changes originate in the samples carrying mutations of relevant genes involved in the methylation process. High-resolution heatmaps for all the chromosomes are provided in the supplementary materials.

## 5. Conclusions

Results to date support a hypothesis for the existence of genome-wide discriminatory information patterns of CDM originating by organismal regulatory responses to environmental variation. Evidence is compelling for hotspots of methylation change. These hotspots are observed on heatmaps as chromosome landmarks located at non-random GRs. Likewise, hotspots of mutational changes were observed as chromosome landmarks. The machine learning approach proposed here permits the detection, throughout feature selection and feature extraction algorithms, of subsets of GFs that carry discriminatory power to discern between individual and between individual subpopulations. The selected GFs were used in the machine learning classifications of methylomes and genomic-mutational variations of *Arabidopsis* ecotypes into groups. Results indicate a strong association between the topologies (numerical taxonomy) of the structured populations of *Arabidopsis* ecotypes based on *I_R_* and *LC_R_*. These results, together with further evaluation of the statistical-physical relationship between SNPs and methylation changes, suggest that divergences originating during the natural mutational process in a structured population are probably influenced by regulatory methylation mechanisms.

## Figures and Tables

**Table 1:** Performance of the classifications presented in Figure 6.

Sample ^1^	Classifier	Accuracy Mean	2.5% Quantile	97.5% Quantile
CG ecotypes (2482 DIRs)	AUC + PCA + LDA	93.08352	88.05678	97.4359
	AUC + PCA + SVM	93.52517	91.83673	95.2381
	AUC + SVM	96.42381	95.91837	96.59864
SNP ecotypes (2590 DIRs)	AUC + LDA	90.85758	85.42125	95.89744
	AUC + PCA + SVM	95.01642	94.02985	96.26866
	AUC + SVM	95.77007	95.23810	95.91837

^1^: 1000 ten-fold cross-validations were performed for each classifier; AUC: Area under ROC curve; PCA: Principal component analysis; SVM: Support vector machine. LDA: Linear discriminant analysis.

**Table 2:** Performance of the classifications presented in Figure 8.

Sample a	Classifier	Accuracy Mean	2.5% Quantile	97.5% Quantile
CG mutants (all regions)	PCA + LDA	93.81872	92.76714	94.85746
	PCA + SVM	94.37882	90.58824	96.47059
CG mutants (9428 DIRs)	PCA + LDA	83.46161	81.47742	85.34079
	SVM	87.83059	84.70588	89.44118
	PCA + SVM	92.77412	89.41176	95.29412
CHG mutants (all regions)	PCA + LDA	95.38752	94.16575	96.57823
	PCA + SVM	97.10741	93.82716	98.76543
CHG mutants (9428 DIRs)	PCA + LDA	93.77919	91.94159	95.3213
	SVM	89.62963	85.18519	92.59259
	PCA + SVM	96.31970	94.94312	97.60015

**Table 3:** Gene set enrichment analysis. This shows the results obtained in the enrichment analysis of 477 gene found in the selected GFs. The *p*-values for each statistical test are given. The table is limited to the GO terms with a *p*-value <0.1 in at least one of the test results obtained from “runTest” function in the “topGO” R package [19] (see also Table S1).

GO.ID	Term (Short Description)	Classic KS Ties ^a^	elimF ^a^	Fisher ^a^
GO:0008150	biological process	0.007	< 0.001	–
GO:0071704	organic substance metabolic process	0.348	0.0015	–
GO:0044238	primary metabolic process	0.164	0.0027	–
GO:0044237	cellular metabolic process	0.305	0.0029	–
GO:0044763	single-organism cellular process	0.246	0.0059	–
GO:0009058	biosynthetic process	0.497	0.0091	–
GO:1901576	organic substance biosynthetic process	0.442	0.0116	–
GO:0050896	response to stimulus	0.068	0.0139	–
GO:0043170	macromolecule metabolic process	0.083	0.0139	–
GO:0044249	cellular biosynthetic process	0.54	0.0198	–
GO:0044260	cellular macromolecule metabolic process	0.129	0.0209	–
GO:0065007	biological regulation	0.811	0.0296	–
GO:0006807	nitrogen compound metabolic process	0.333	0.0332	–
GO:0044710	single-organism metabolic process	0.748	0.0371	–
GO:0034641	cellular nitrogen compound metabolic process	0.629	0.0415	<0.001
GO:1901360	organic cyclic compound metabolic process	0.233	0.0439	–
GO:0050789	regulation of biological process	0.913	0.0439	–
GO:0006725	cellular aromatic compound metabolic process	0.327	0.0548	<0.001
GO:0046483	heterocycle metabolic process	0.294	0.0611	<0.001
GO:0050794	regulation of cellular process	0.889	0.0682	<0.001
GO:0009059	macromolecule biosynthetic process	0.369	0.0759	<0.001
GO:0006139	nucleobase-containing compound metabolic	0.202	0.0845	<0.001
GO:0034645	cellular macromolecule biosynthetic process	0.569	0.0892	<0.001
GO:0032502	developmental process	0.046	0.094	–
GO:0006950	response to stress	0.094	0.094	<0.001
GO:0044767	single-organism developmental process	0.029	0.0991	<0.001

^a^ Parameter setting for the topGO function “runTest”. Classic KS ties: “classic” algorithm with Kolmogorov–Smirnov test. elimF: algorithm with Fisher test. Fisher: Gene enrichment is based on a Fisher exact test for the set of genes found in the corresponding GO term in respect to the set of all genes found in selected genomic features. The p-values derived from the corresponding tests are given. The p-values lesser than 0.001 are written as “< 0.001”, while the symbol “-” indicates that not testing was performed.

**Table 4:** Genes within selected GFs from the GO:0006950 (response to abiotic stress, response to biotic stress). The enrichment analysis is based on a Fisher exact test yield *p*-value <0.001 (see Table S4).

Gene.ID	Name (Short Description)	$\chi_{LC_{R}}^{2}/\chi_{I_{R}}^{2}$ ^a^
AT1G16540	ABA3; K15631 molybdenum cofactor sulfurtransferase (C:2.8.1.9)	1.11
AT1G19180	JAZ1; K13464 jasmonate ZIM domain-containing protein	0.98
AT1G19480	K01247 DNA-3-methyladenine glycosylase II (EC:3.2.2.21)	1.02
AT1G28480	GRX480; glutaredoxin-GRX480	1.54
AT1G31812	ACBP6; acyl-CoA-binding protein 6	0.98
AT1G54610	K08819 cyclin-dependent kinase 12/13 (EC:2.7.11.22 2.7.11.23)	0.81
AT1G69940	PPME1; pectinesterase PPME1; K01051 pectinesterase (EC:3.1.1.11)	0.55
AT1G76500	SOB3; AT-hook motif nuclear localized protein 29	0.42
AT2G05990	MOD1; K00208 enoyl-[acyl-carrier protein] reductase I (EC:1.3.1.9 1.3.1.10)	0.77
AT2G11000	MAK10; MAK10-like protein	1.07
AT2G28660	chloroplast-targeted copper chaperone protein	1.43
AT2G30750	CYP71A12; cytochrome P450 71A12; K00517 (EC:1.14.-.-)	1.13
AT2G32660	RLP22; receptor like protein 22	1.26
AT2G34390	NIP2;1; aquaporin NIP2-1; K09874 aquaporin NIP	1.66
AT2G43620	chitinase family protein; K01183 chitinase (EC:3.2.1.14)	0.76
AT2G47000	ABCB4; auxin efflux MDR4; K05658 (EC:3.6.3.44)	1.17
AT3G21860	SK10; SKP1-like protein 10; K03094 S-phase kinase-associated protein 1	0.58
AT3G28360	PGP16; ABC transporter B family member 16; K05658 (EC:3.6.3.44)	0.81
AT3G32920	DNA repair protein recA homolog 4; K03553 recombination protein RecA	1.01
AT3G44110	ATJ3; chaperone protein dnaJ 3; K09503	1.1
AT3G44480	RPP1; TIR-NBS-LRR class disease resistance protein	1.01
AT3G45140	LOX2; lipoxygenase 2; K00454 lipoxygenase (EC:1.13.11.12)	1.21
AT3G45260	C2H2-like zinc finger protein	0.96
AT3G46970	PHS2; K00688 starch phosphorylase (EC:2.4.1.1)	1.08
AT3G61220	K15095 (+)-neomenthol dehydrogenase (EC:1.1.1.208)	1.57
AT4G04770	ABC1; ATP binding cassette protein 1; K07033 uncharacterized protein	1.15
AT4G13920	RLP50; receptor like protein 50	0.93
AT4G19840	PP2-A1; protein PHLOEM protein 2-LIKE A1	0.8
AT4G24670	TAR2; K16903 L-tryptophan—pyruvate aminotransferase (EC:2.6.1.99)	1.28
AT4G27410	RD26; NAC transcription factor RD26	0.62
AT5G24360	IRE1-1; protein inositol requiring 1-1	1.52
AT5G38340	TIR-NBS-LRR class disease resistance protein	1.16
AT5G42020	BIP2; Luminal-binding protein 2; K09490 heat shock 70kDa protein 5	1.03
AT5G42540	XRN2; 5’-3’ exoribonuclease 2; K12619 5’-3’ exoribonuclease 2 (EC:3.1.13.-)	1.17
AT5G43810	ZLL; eIF2C Argonaute10; K11593 eukaryotic translation initiation factor 2C	0.94
AT5G44910	Toll-Interleukin-Resistance domain-containing protein	0.9
AT5G51630	TIR-NBS-LRR class disease resistance protein	0.9

^a^ Chi-squared statistics estimated for GR selected features where the annotated protein-coding gene is located.

## Supplementary Materials

Supplementary materials can be found at http://www.mdpi.com/1422-0067/17/6/938/s1.

## Abbreviations

AUC	Area under ROC curve
CDF	Cumulative distribution function
CDM	Cytosine DNA methylation
DI	Discriminatory information
DMR	differentially methylated region
FGM	Farlie–Gumbel–Morgenstern
LDA	Linear discriminant analysis
LD	Linear discriminant
GF	genomic feature
GR	genomic region
PC	Principal component
PCA	PC analysis
ROC	receiver operating characteristic
SVM	Support vector machine
SNP	Single nucleotide polymorphism
CMT3	CHROMOMETHYLASE 3, a chromomethylase involved in methylating cytosine residues at non-CG sites
DDM1	decreased dna methylation 1
DRM(n)	domains rearranged methyltransferase n, e.g., DRM1
DCL(n)	DICER-LIKE n, e.g., DICER-LIKE 4 (DCL4)
HEN1	a methyltransferase that methylates miRNAs and siRNAs
IDN2	involved in *de novo* 2
IDNL(n)	involved in *de novo* 2 (IDN2)—LIKE n, e.g., IDNL1
MET1	methyltransferase 1
sdg2	mutant of the SET DOMAIN PROTEIN 2, a histone-lysine N-methyltransferase (H3-K4 specific)
RDR(n)	RNA-dependent RNA polymerases, e.g., RDR1
SUVH(n)	histone 3 lysine 9 (H3K9) specific methyltransferase involved in the maintenance of DNA methylation
SUVH4 is also called KRYPTONITE (KYP); suvh456 denote the triple mutant suvh4, suvh5, and suvh6	
UPGMA	Stands for Unweighted Pair Group Method with Arithmetic Mean
VIM(n)	variant in methylation family protein, e.g., VIM1
ddc	triple mutant drm1 drm2 and cmt3
