# Supplementary material for: Genome-Wide Discriminatory Information Patterns of Cytosine DNA Methylation
Source: Int J Mol Sci. 2016 Jun 17;17(6):938. doi: 10.3390/ijms17060938 (PMC4926471; doi:10.3390/ijms17060938)
Supplement: Supplementary file 1 [file ijms-17-00938-s001.pdf]

Supplementary Materials: Genome-Wide Discriminatory Information Patterns of Cytosine DNA Methylation

Robersy Sanchez and Sally A. Mackenzie

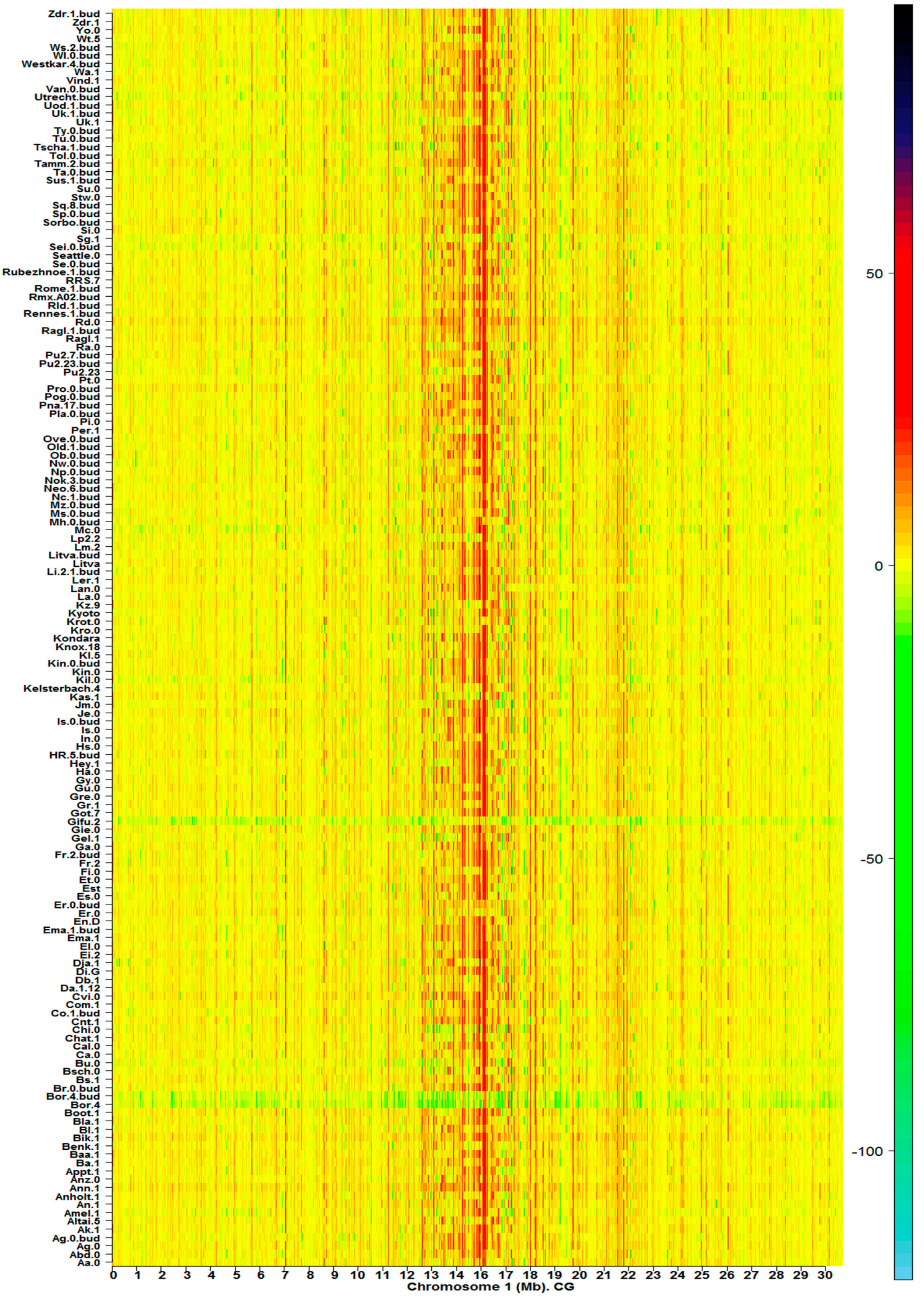

Figure S1. Heatmap of I_R for CG methylation context in chromosomes 1, built for the 151 *Arabidopsis thaliana* ecotypes [14].

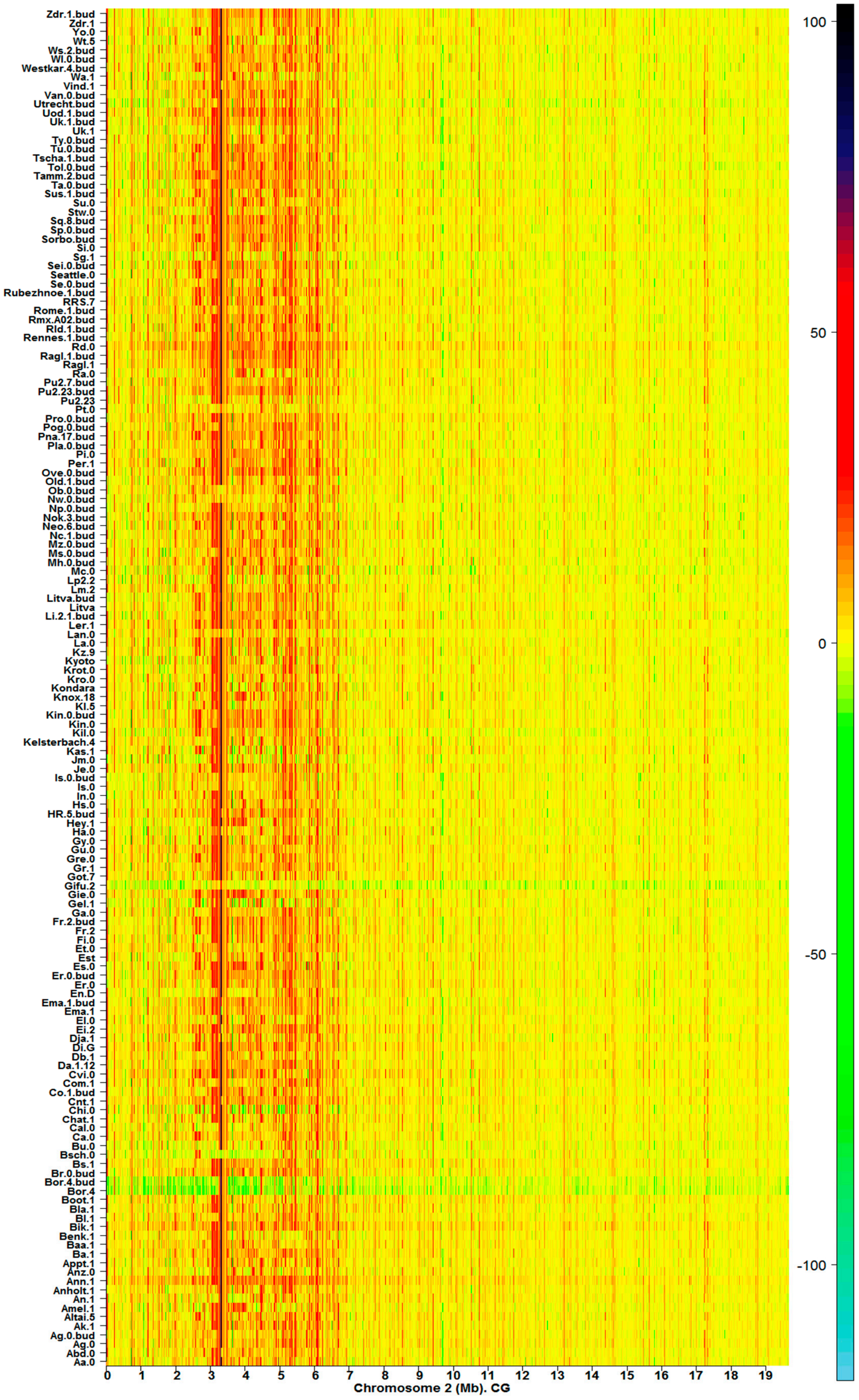

Figure S2. Heatmap of I_R for CG methylation context in chromosomes 2, built for the 151 *Arabidopsis thaliana* ecotypes [14]

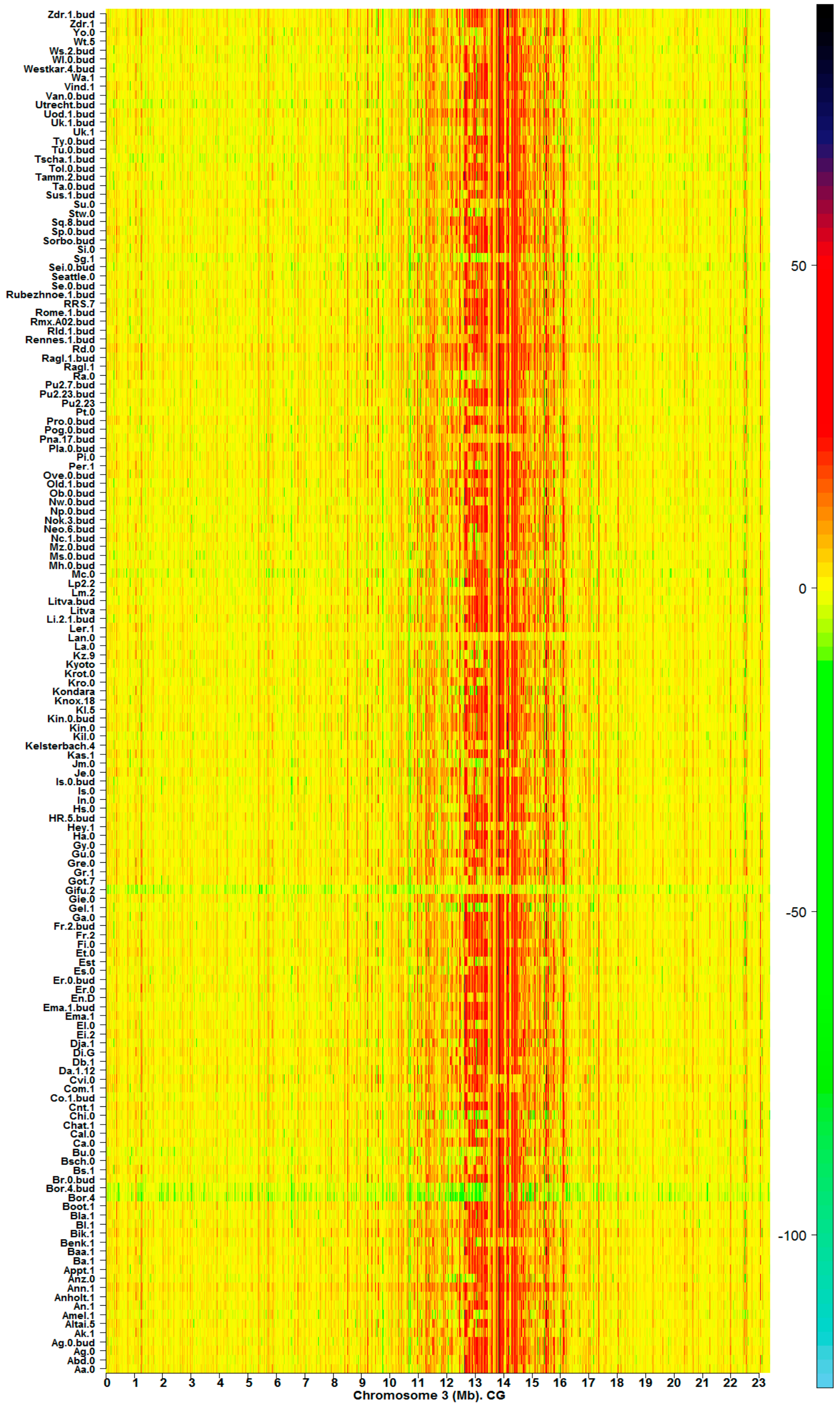

Figure S3. Heatmap of I_R for CG methylation context in chromosomes 3, built for the 151 *Arabidopsis thaliana* ecotypes [14]

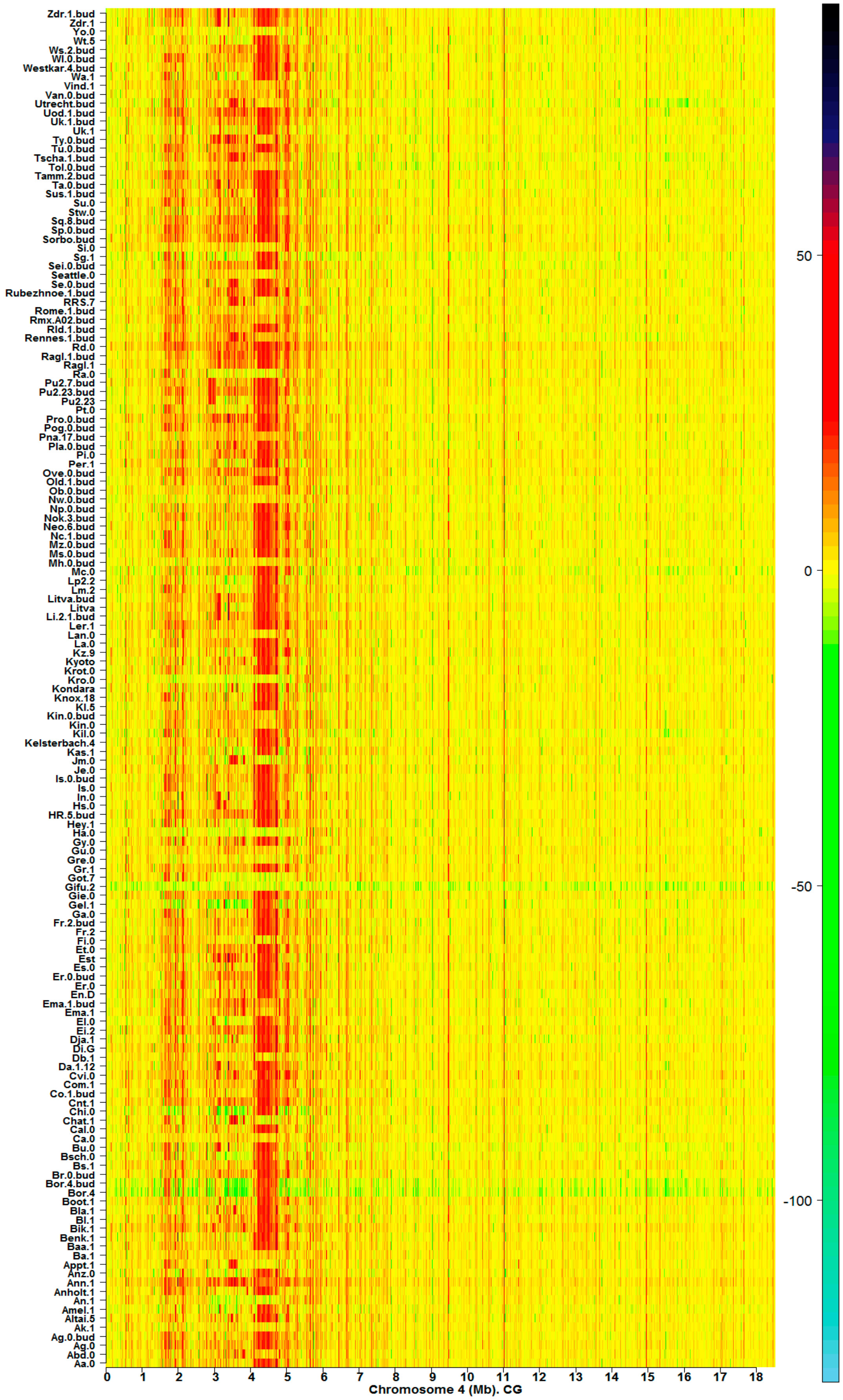

Figure S4. Heatmap of I_R for CG methylation context in chromosomes 4, built for the 151 *Arabidopsis thaliana* ecotypes [14]

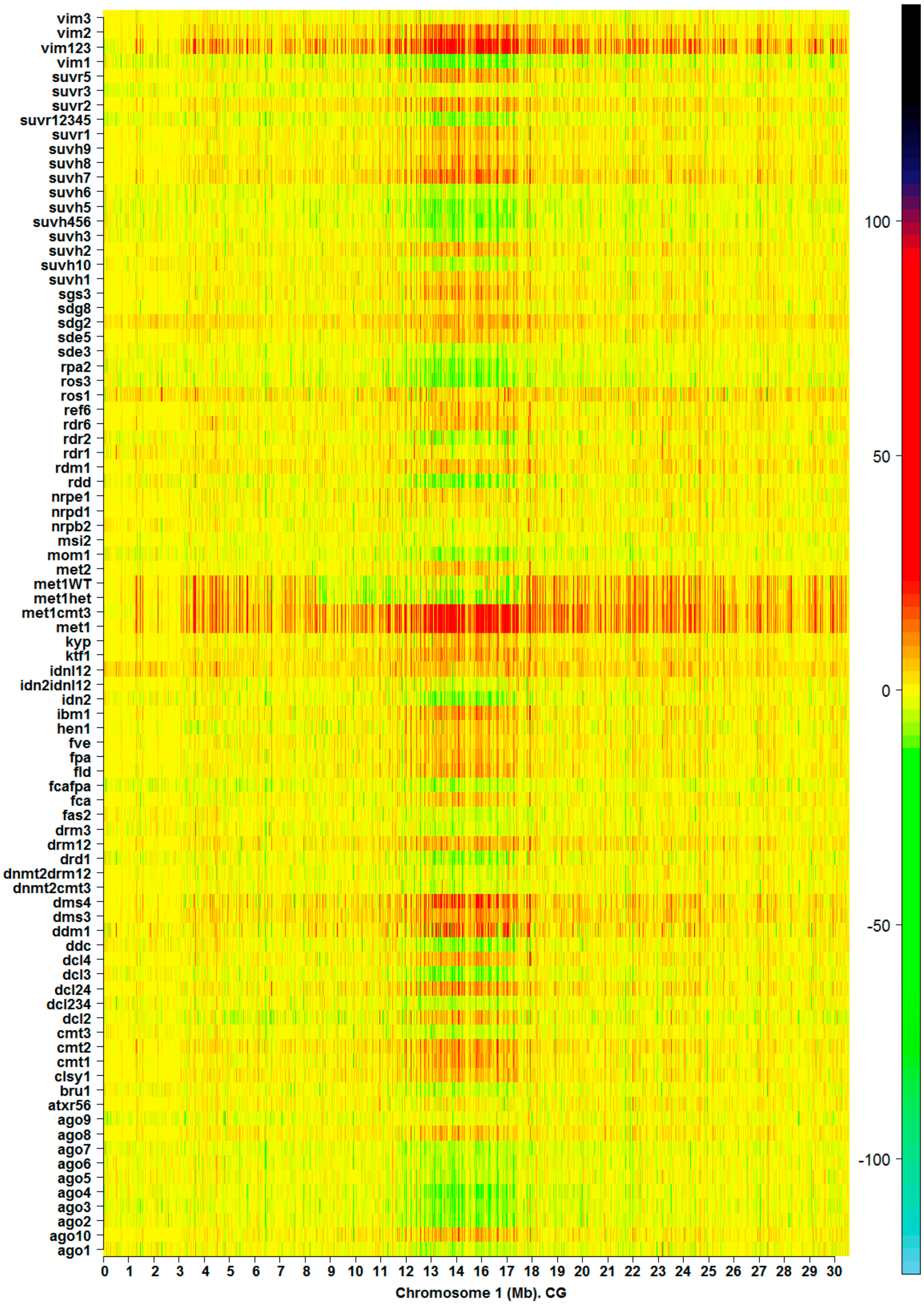

Figure S5. Heatmap of I_R for CG methylation context in chromosomes 1 built for 85 silencing mutants in Arabidopsis thaliana [15].

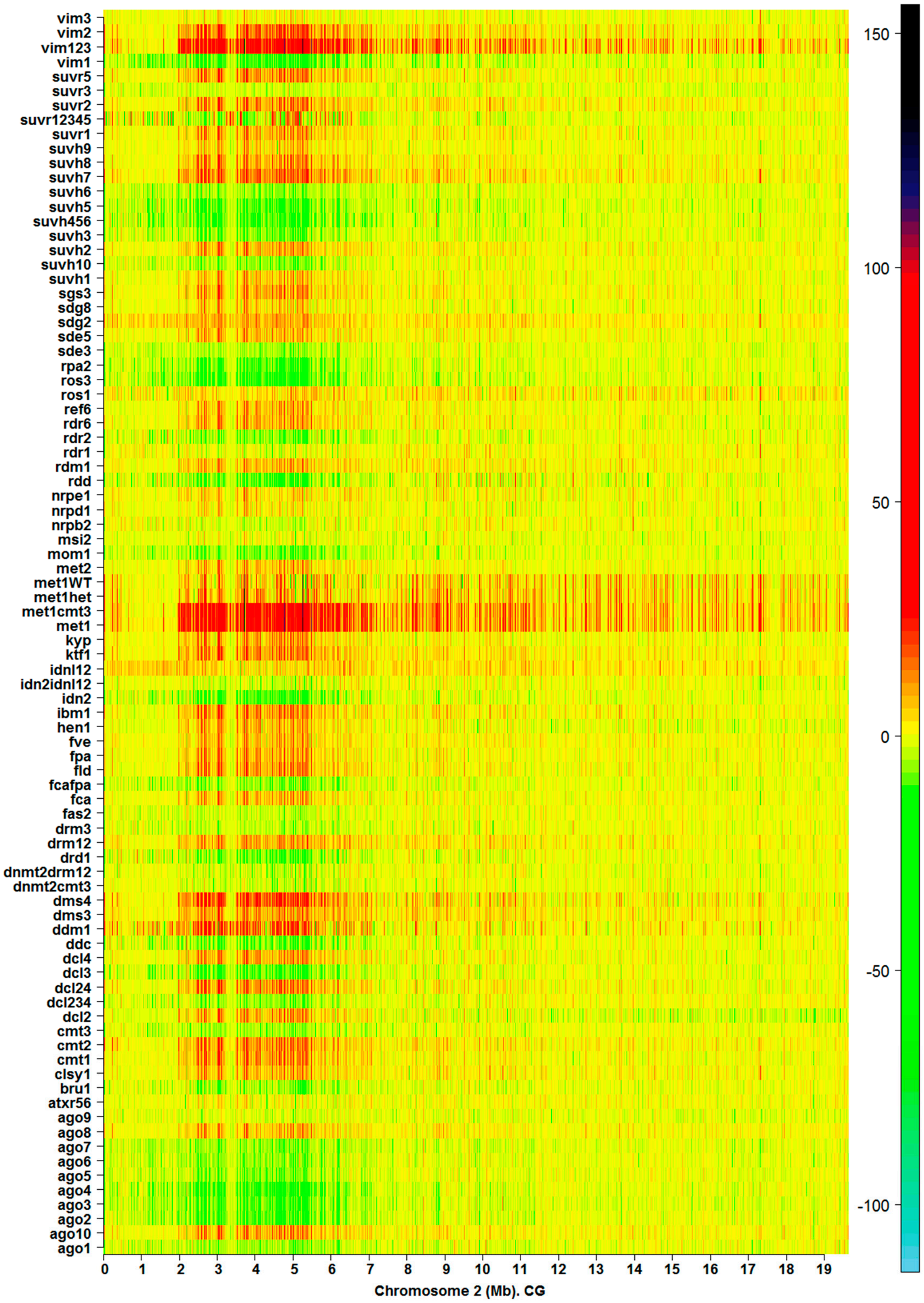

Figure S6. Heatmap of I_R for CG methylation context in chromosomes 2 built for 85 silencing mutants in Arabidopsis thaliana [15].

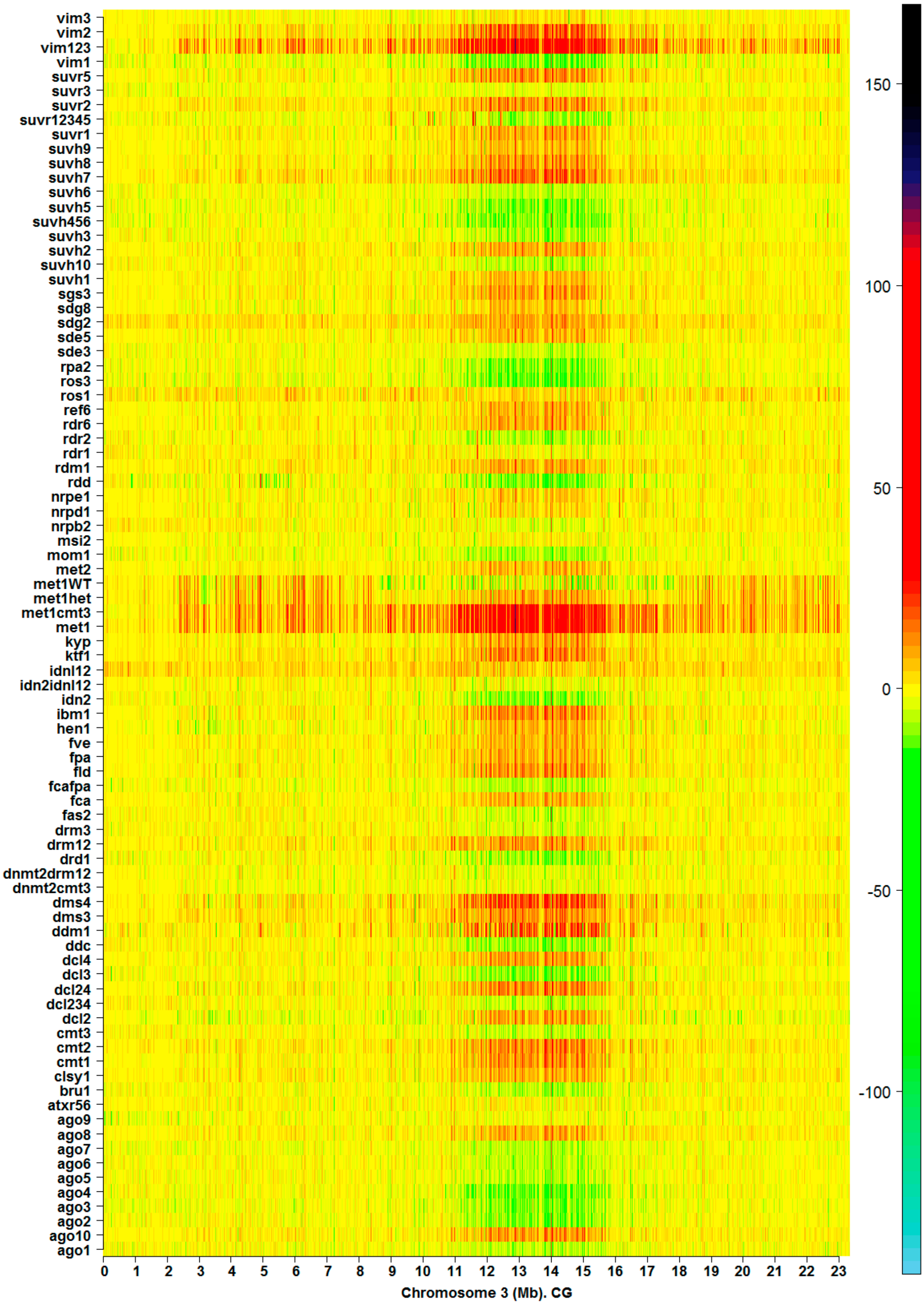

Figure S7. Heatmap of I_R for CG methylation context in chromosomes 3 built for 85 silencing mutants in Arabidopsis thaliana [15].

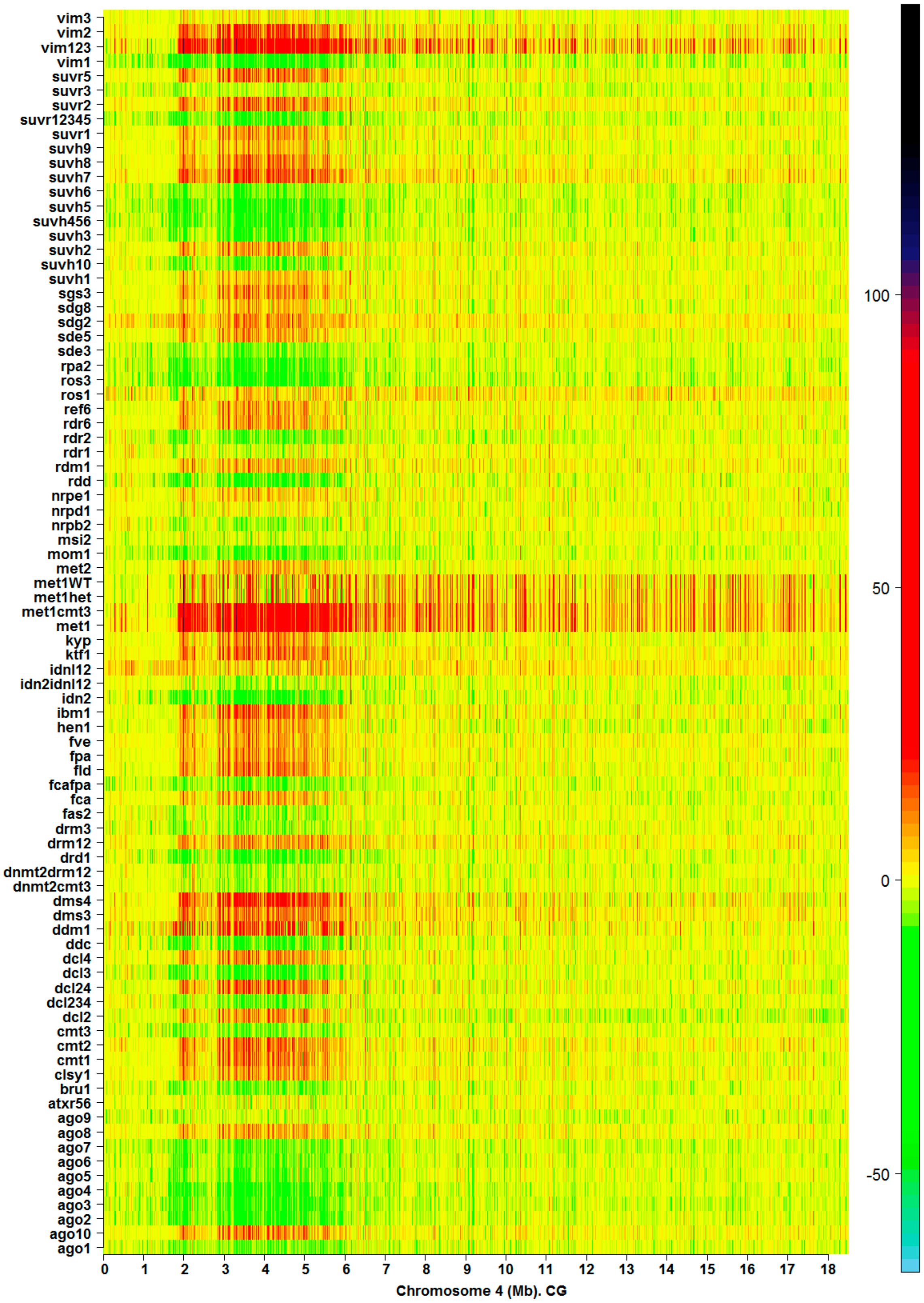

Figure S8. Heatmap of I_R for CG methylation context in chromosomes 4 built for 85 silencing mutants in Arabidopsis thaliana [15].

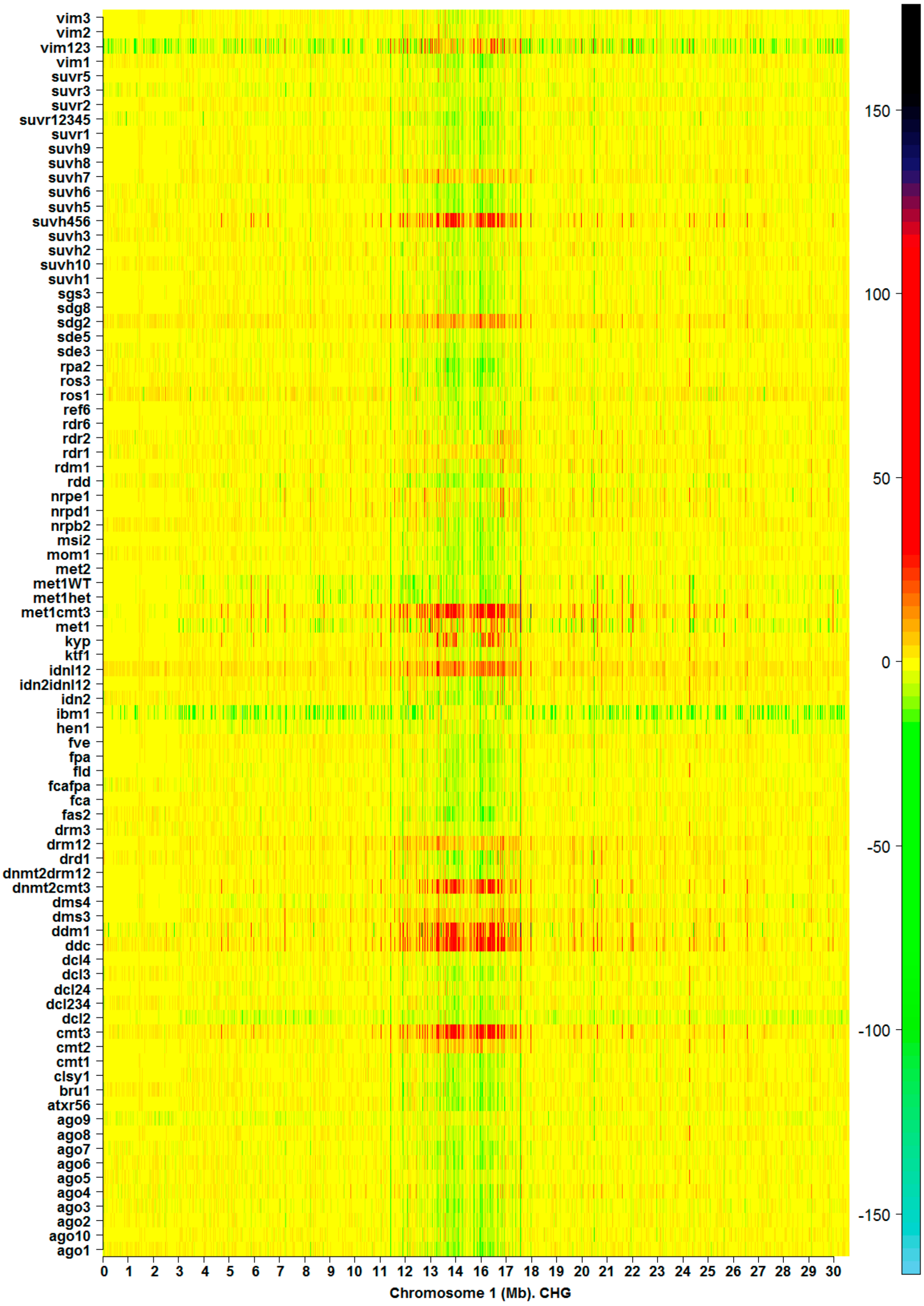

Figure S9. Heatmap of I_R for CHG methylation context in chromosome 1 built for the 85 silencing mutants [15].

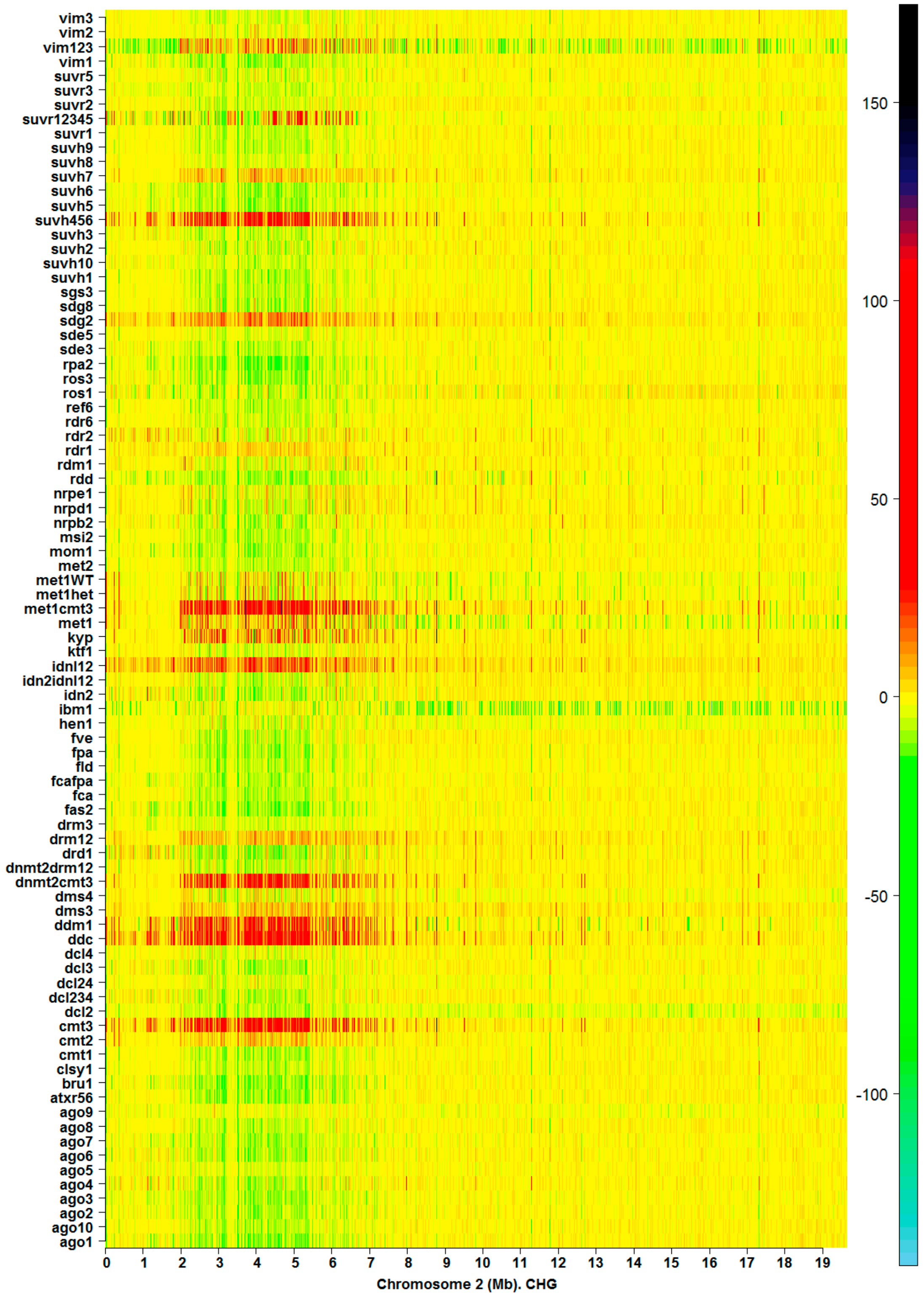

Figure S10. Heatmap of I_R for CHG methylation context in chromosome 2 built for the 85 silencing mutants [15].

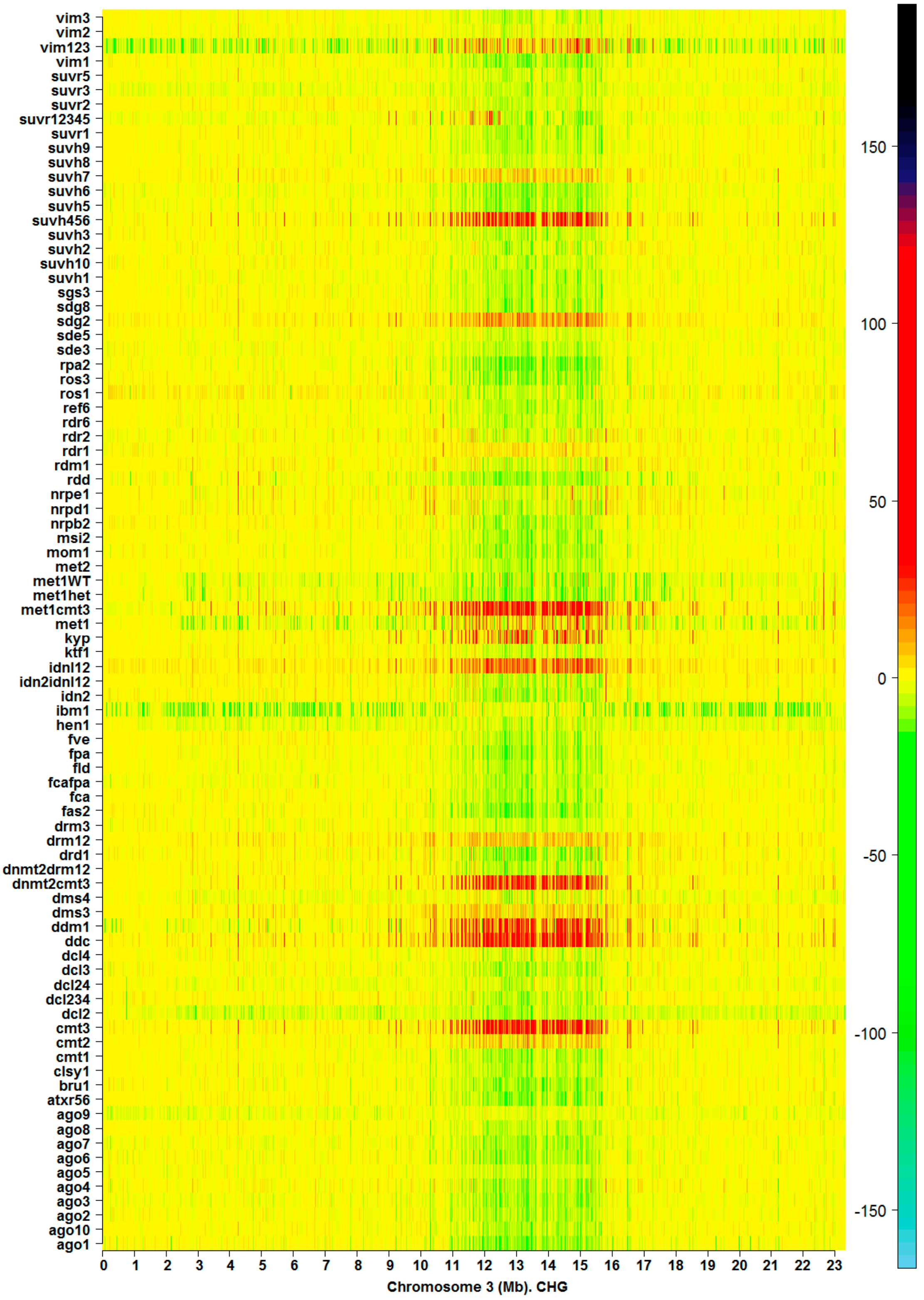

Figure S11. Heatmap of I_R for CHG methylation context in chromosome 3 built for the 85 silencing mutants [15].

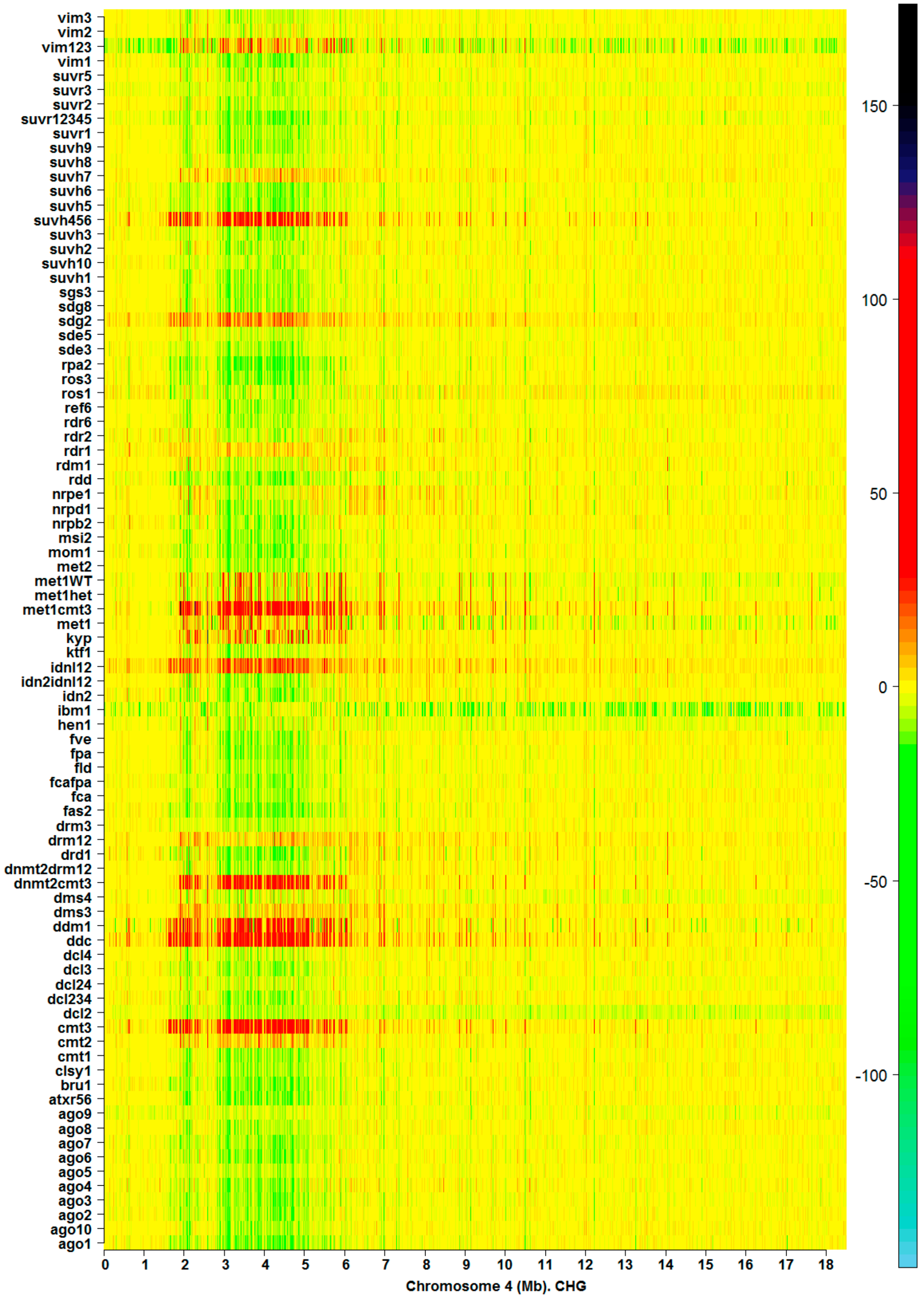

Figure S12. Heatmap of I_R for CHG methylation context in chromosome 4 built for the 85 silencing mutants [15].

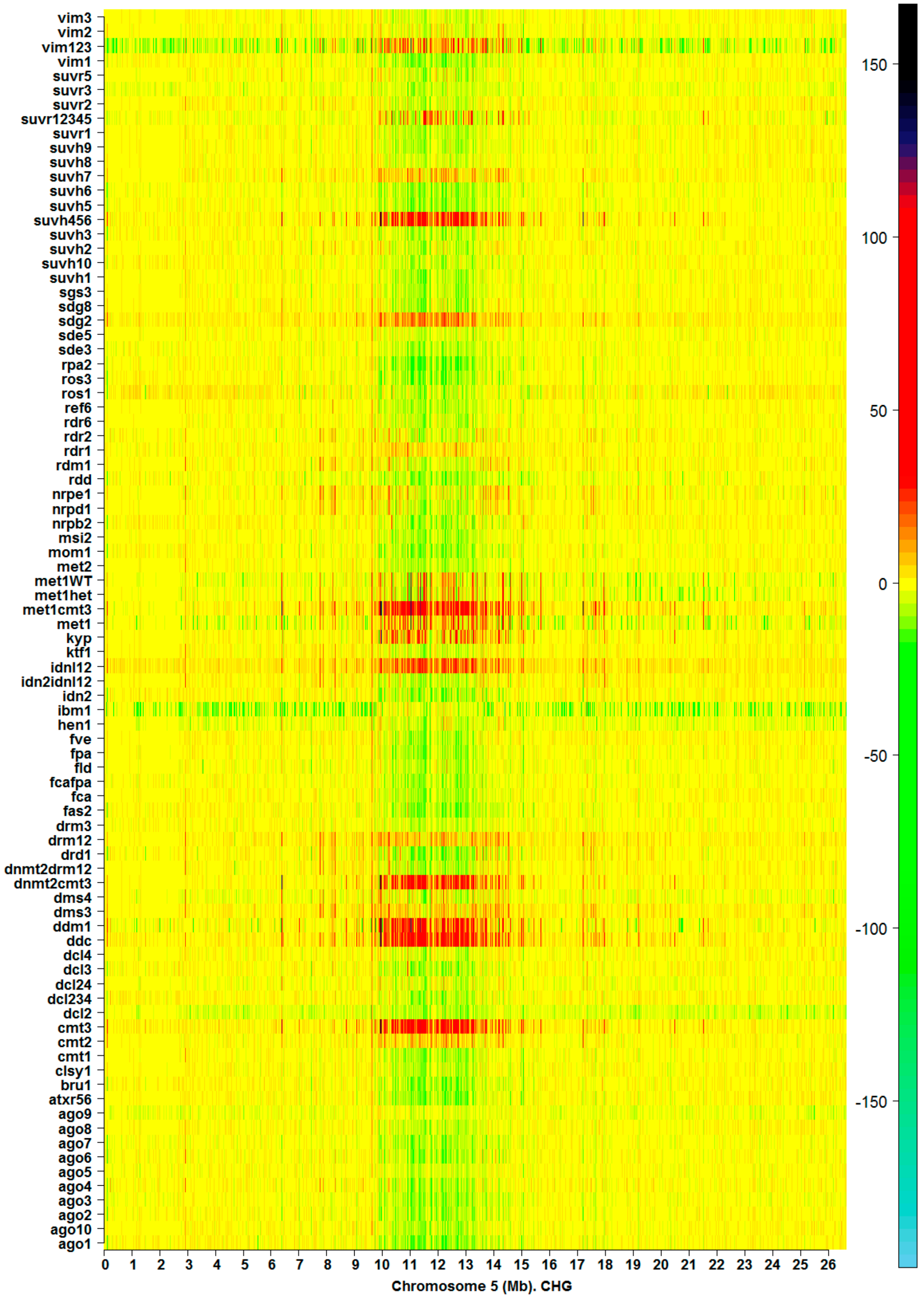

Figure S13. Heatmap of I_R for CHG methylation context in chromosome 5 built for the 85 silencing mutants [15].

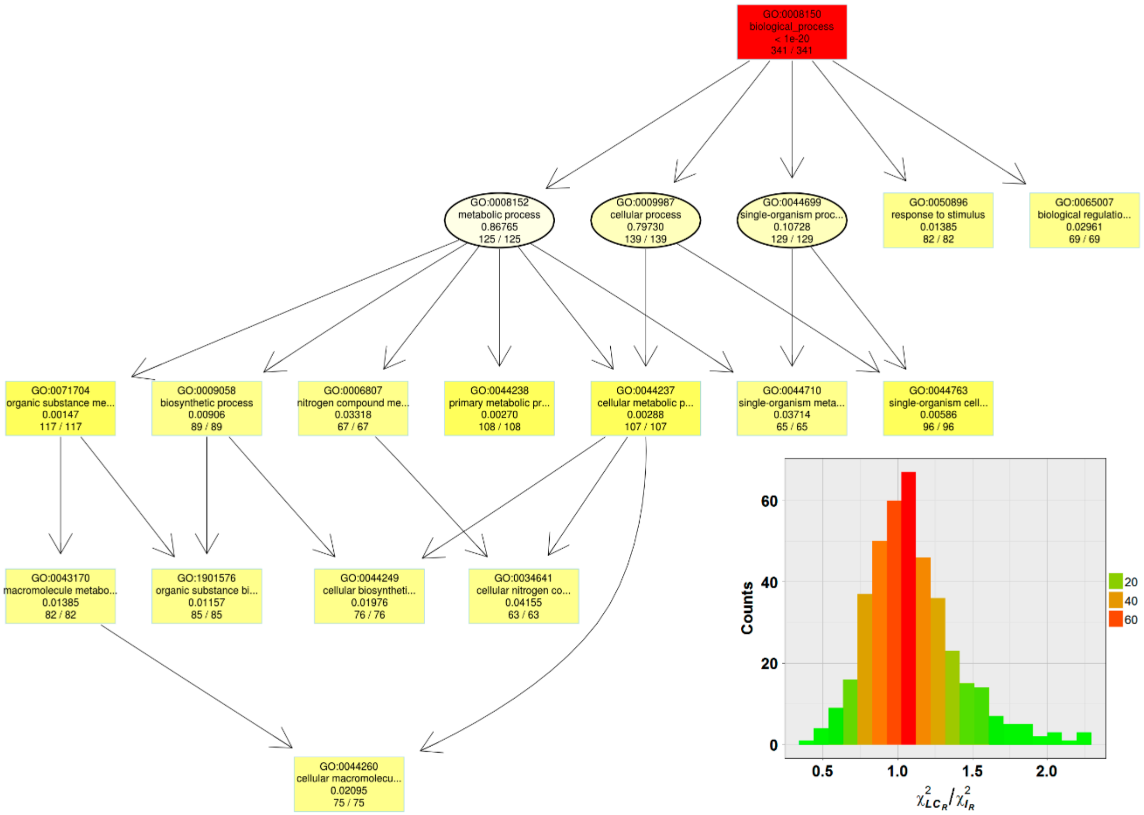

Figure S14. GO enrichment analysis of the genes within the selected GFs based on Chi-square statistics. The significant nodes are represented as rectangles and only the subgraph induced by the first 15 significant nodes is shown. The subplot shows the distribution of the rate $\chi^2_{LC_R}/\chi^2_{I_R}$ of the Chi-squared statistics estimated for GFs selected where the 477 annotated protein-code genes are located. The bootstrap Kolmogorov–Smirnov test does not reject normality rate $\chi^2_{LC_R}/\chi^2_{I_R}$ and a Student's *t*-test indicates that the mean is significantly greater than 1 (although quantitatively not too much greater than 1, since the expected mean inferred for this population is around 1.1.

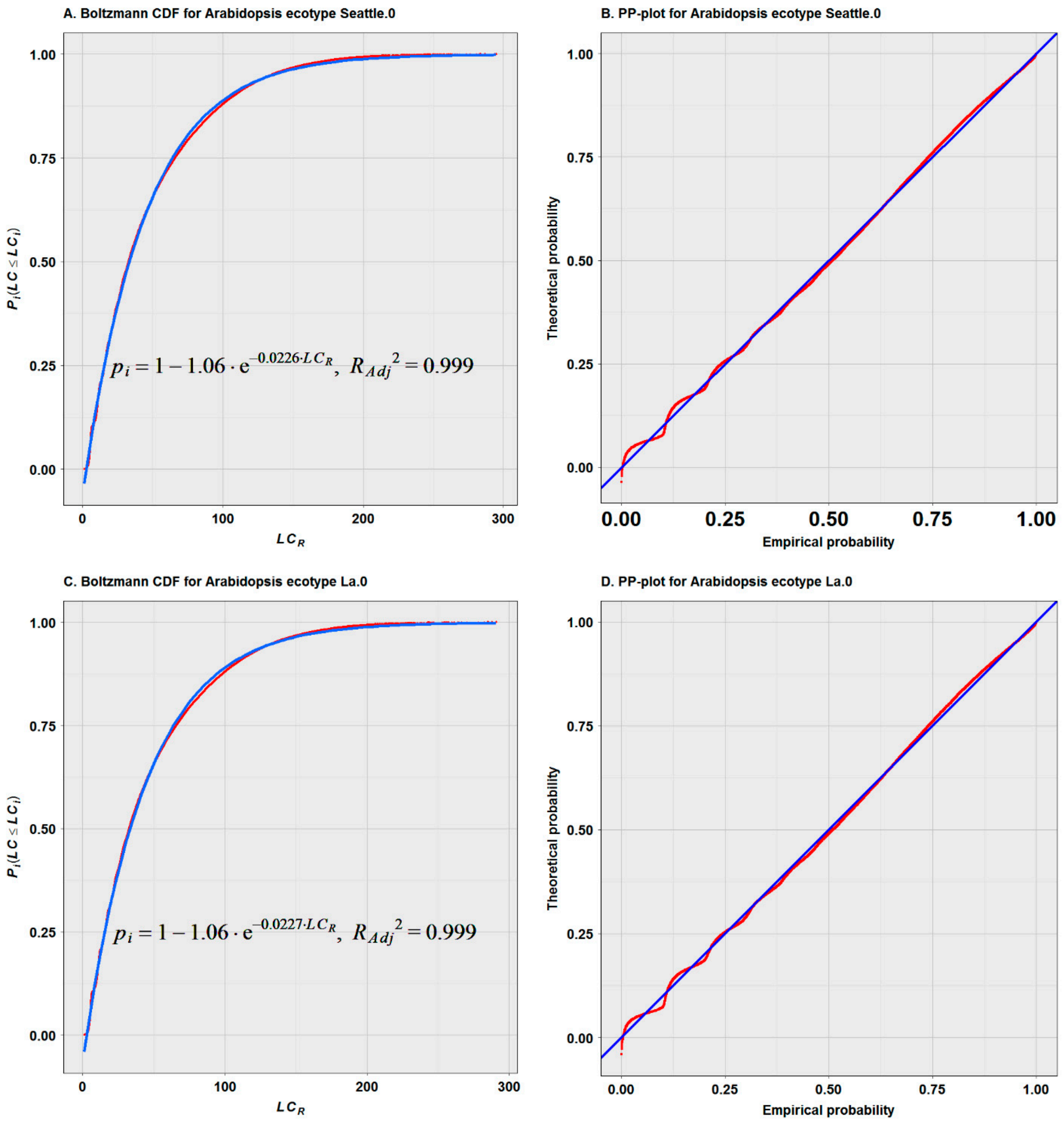

Figure S15. Non-linear regression fit of Equation (4) for *Arabidopsis* ecotypes Seattle.0 and La.0. (**A**); CDF and PP-plot *Arabidopsis* ecotype Seattle.0. (**B**,**C**); CDF and PP-plot *Arabidopsis* ecotype La.0. (**D**). In panels **A** and **C**, the blue and red lines denote the fitted and experimental probability values, respectively; In panels **B** and **D**, the red lines correspond to the graph: theoretical probability (y) versus empirical probability (x), and the blue lines are only the reference straight line $x = y$, which correspond to the ideal/perfect fit.

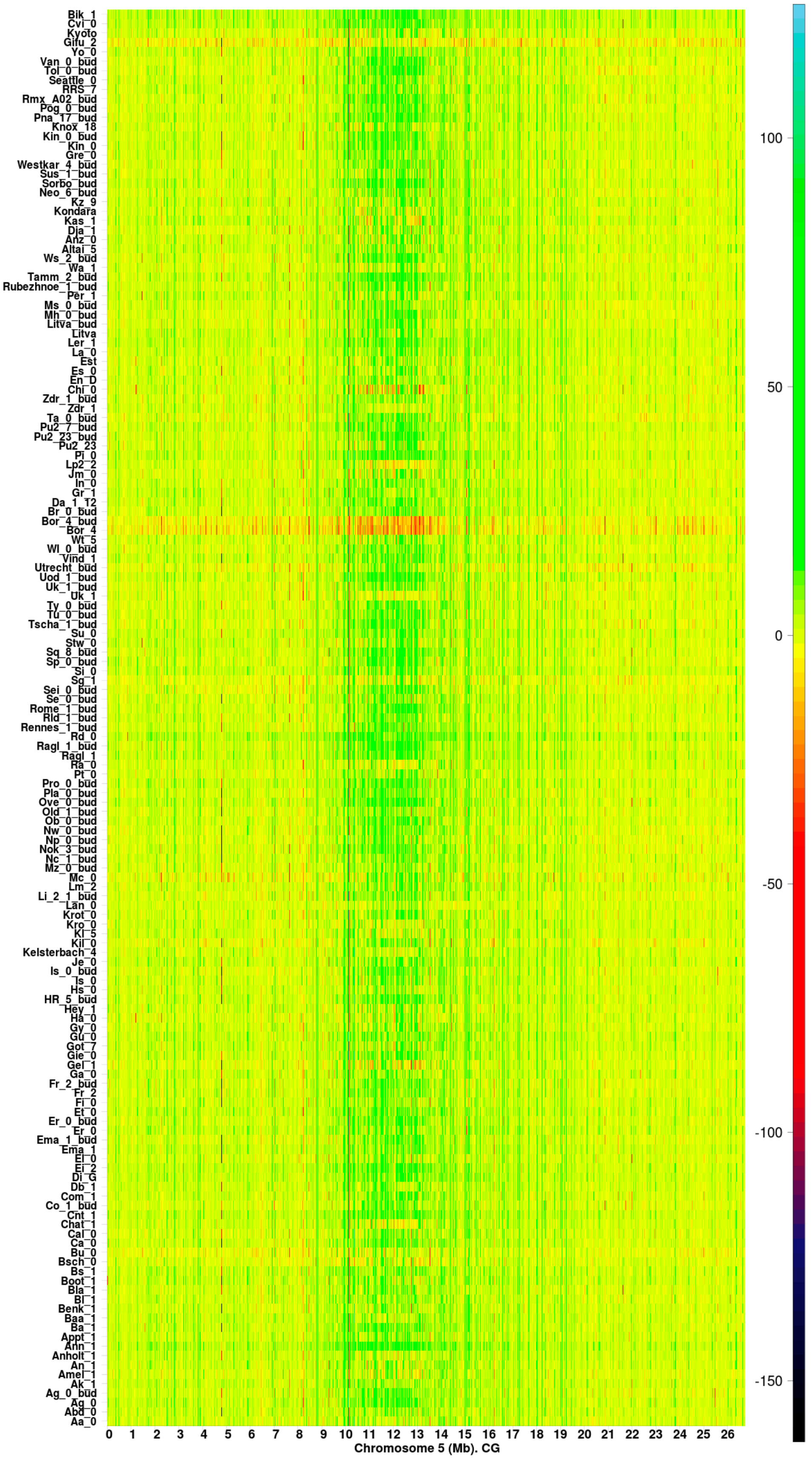

Figure S16. Heatmap for I_R in the CG methylation context along chromosome 5 from 151 *Arabidopsis thaliana* ecotypes. The samples are the same as in Figure 1, but with an inverted color scale equivalent to a photographic negative; the maximum and minimum correspond to sky blue and black, respectively.
